# Alchemizing earth's legacy: Bismuth-engineered humic nanoparticles for IBD theranostics through mitochondrial anti-inflammation and sustained intestinal delivery

**DOI:** 10.1016/j.mtbio.2025.101948

**Published:** 2025-06-06

**Authors:** Ganglin Wang, Ziwei Wang, Lin Liu, Yejing Zhu, Jiali Zhong, Jiayi Zhang, Lingling Wang, Chenguo Zheng, Wei Li

**Affiliations:** a Key Laboratory of Laboratory Medicine, Ministry of Education of China, Zhejiang Provincial Key Laboratory of Medical Genetics, School of Laboratory Medicine and Life Sciences, Wenzhou Medical University, Wenzhou, 325035, China; b Department of Coloproctology, The Second Affiliated Hospital and Yuying Children's Hospital of Wenzhou Medical University, Wenzhou, 325000, China

**Keywords:** Inflammatory bowel diseases, BiHANs, ROS scavenging, Gut microbiota modulation, CT imaging

## Abstract

Humic acid (HA), a complex organic substance naturally present in soil, peat, and coal, is traditionally referred to as the extract of *"Wujinshi"* (乌金石) in Chinese medicine. It is renowned for its potent anti-inflammatory, analgesic, and blood circulation-promoting properties. However, its mechanisms and delivery methods require further exploration. We developed BiHANs, a novel nanoparticle combining bismuth and HA, which exhibits excellent biocompatibility and anti-inflammatory effects *in vitro*. Orally administered BiHANs accumulate at inflammatory sites in the colon of DSS-induced IBD mice, as confirmed by CT imaging, and prolong intestinal retention. They significantly alleviate acute and chronic intestinal damage by suppressing inflammatory responses, modulating gut microbiota, and targeting macrophage mitochondria to inhibit NF-κB-driven cytokine production. BiHANs also maintain mitochondrial homeostasis and exert antioxidative effects. Furthermore, their performance in CT imaging highlights potential as a theranostic agent. This study demonstrates BiHANs as a promising platform for targeted IBD therapy and diagnosis, combining anti-inflammatory efficacy with imaging capabilities.

## Highlights

• Metal-phenolic engineered BiHANs achieve oral colon-targeted delivery.
• Prolonged gut retention enhances localized anti-inflammatory efficacy.
• Mitochondrial ROS scavenging suppresses NF-κB inflammation.
• Theranostic BiHANs integrate therapy and CT imaging.
• Microbiota reprogramming synergizes with IBD treatment.

## Graphical abstract

**Figure:**
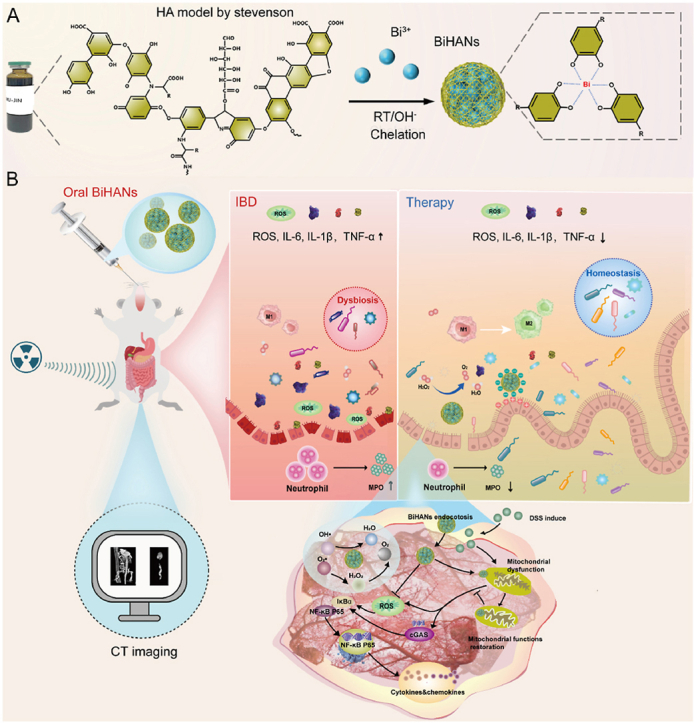

## 1 Introduction

Inflammatory bowel disease (IBD), encompassing Crohn's disease and ulcerative colitis, is a chronic and relapsing inflammatory disorder of the gastrointestinal tract [1,2]. It is characterized by dysregulated immune responses, oxidative stress, and gut microbiota dysbiosis [[3], [4], [5]]. Despite significant advancements in therapeutic strategies—such as anti-inflammatory drugs, immunosuppressants, and biologics—the management of IBD remains challenging [[6], [7], [8]]. This is due to the complexity of its pathogenesis and the limited efficacy or potential side effects of current treatments [9,10]. Therefore, there is an urgent need for novel therapeutic approaches that can address multiple aspects of IBD pathology, including inflammation, oxidative stress, and microbial imbalance.

Traditional Chinese medicine (TCM) has long been used to treat gastrointestinal disorders, including IBD, with promising clinical outcomes [[11], [12], [13], [14], [15], [16], [17]]. Unlike single-target therapies, TCM typically employs multi-component formulations that synergistically modulate various pathological processes, such as inflammation, oxidative stress, and immune dysregulation [11]. This multi-target approach aligns well with the complex nature of IBD, making TCM a valuable resource for developing new therapeutic strategies [12]. However, the poor solubility, stability, and bioavailability of many TCM components often limit their clinical application, highlighting the need for innovative formulation strategies.

Humic acid (HA) is a naturally occurring organic substance derived from the decomposition of plant and microbial matter over centuries [18]. It has been documented in the Compendium of Materia Medica (Ben Cao Gang Mu), named *Wujinshi* (a type of lignite) under the category of mineral-based drugs [19]. It is a complex mixture of bioactive components, including phenolic compounds, carboxyl groups, hydroxyl groups, and aromatic structures, which collectively contribute to its diverse pharmacological properties [[20], [21], [22], [23]]. Interestingly, HA can be viewed as a natural integration of multiple plant-derived components, as it originates from the breakdown of various plant materials, such as lignin, cellulose, and other organic matter. This decomposition process results in a unique amalgamation of bioactive molecules that may encapsulate the therapeutic essence of numerous plants [24]. In this sense, HA represents a natural "integration" of plant-based compounds, akin to a complex herbal formulation found in traditional medicine systems. Its multi-component nature enables HA to exhibit a wide range of biological activities, including anti-inflammatory, antioxidant, and microbiota-modulating effects, making it a promising candidate for treating complex diseases such as IBD [25]. By leveraging the inherent complexity of HA, we can explore its potential as a novel therapeutic agent that harnesses the synergistic effects of its integrated plant-derived components. Despite its therapeutic benefits, the clinical application of HA has been hindered by its poor solubility and lack of effective delivery systems, which limit its bioavailability and therapeutic efficacy [26]. Therefore, developing advanced formulations of HA that enhance its stability, bioavailability, and targeted delivery is crucial for maximizing its therapeutic potential.

Recent advances in nanotechnology have demonstrated that complex natural components can self-assemble with metal ions to form functional nanoparticles [[27], [28], [29], [30]]. Building on this foundation, IBD therapeutics have witnessed transformative innovations: Metal-phenolic coordination strategies now enable precise inflammatory pathway regulation, while multifunctional nanozymes set new benchmarks in concurrent antioxidant and anti-inflammatory action [28,31]. Recent progress further reveals nanoparticle-mediated microbiome restoration and mitochondrial protection as critical interventions for IBD's multi-scale pathology [32,33]. Importantly, emerging bismuth-based contrast agents [34] and sustainable biomaterial designs [35] address long-standing gaps in therapeutic monitoring and clinical translation. These advancements collectively highlight the potential of metal-natural component hybrids as integrated theranostic platforms.

**Scheme 1:**
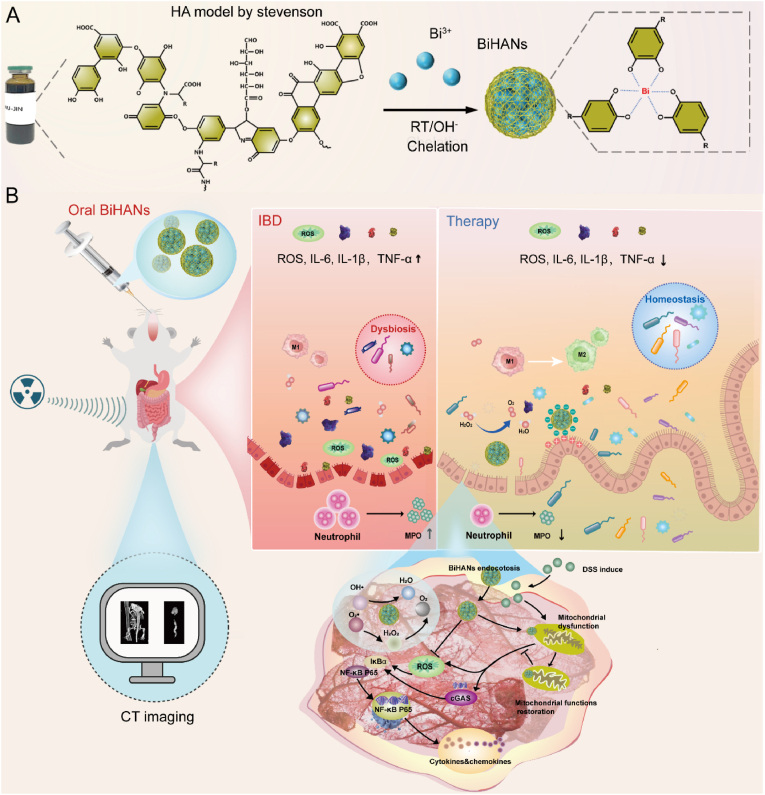
Schematic illustration of the therapeutic mechanism of oral BiHANs for IBD. A) Schematic synthesis of BiHANs. B) Schematic drawing illustrating BiHANs target inflammatory Sites, alleviate IBD via mitochondrial homeostasis restoration and anti-inflammatory effects.

Herein, inspired by this approach, we explored the possibility of combining HA with bismuth, a metal widely used in the treatment of gastrointestinal diseases due to its anti-inflammatory and antimicrobial properties. By leveraging the coordination between HA and bismuth ions (Bi^3+^), we developed bismuth-humic acid nanoparticles (BiHANs) as a novel nanotherapeutic platform for IBD treatment. This study aims to investigate the therapeutic potential of BiHANs in alleviating colitis, with a focus on their antioxidant, anti-inflammatory, and microbiota-modulating effects, as well as their ability to target mitochondria and modulate the NF-κB signaling pathway (see Scheme 1).

## 2 Experimental section/methods

### 2.1 Materials

Humic acids (HA) was purchased from Sigma-Aldrich (USA). Bi(NO_3_)_3_·5H_2_O was purchased from Adamas (China). FeCl_3_·6H_2_O was purchased from Tianjin Heowns Technology Co., Ltd. DAPI, the MTT cell proliferation and cytotoxicity assay kit, cell counting kit-8 (CCK-8), calcein-AM, propidium iodide (PI), the bicinchoninic acid (BCA) kit, and 1-(3-dimethylaminopropyl)-3-ethylcarbodiimide (EDC) were purchased from Beyotime Biotech Inc. (Shanghai, China). Simulated gastric fluid, lipopolysaccharide (LPS), and bismuth standard solution were purchased from Solarbio Technology Co., Ltd. (Beijing, China). Simulated intestinal fluid was purchased from Leagene Biotech Co., Ltd. (Beijing, China). Sulfo Cy5.5 NH_2_ was purchased from Xi'an Ruixi Biotech Co., Ltd. 2,2-Diphenyl-1-picrylhydrazyl (DPPH) was purchased from TargetMol Biotech Co., Ltd. (Shanghai, China). DCFH-DA was purchased from Sigma-Aldrich (USA). Hydrogen peroxide was purchased from G-Clone Biotech Co., Ltd. (Beijing, China). Sulfo-NHS was purchased from Shanghai Macklin Biochemical Co., Ltd. The JC-1 assay kit and Ioversol were purchased from Aladdin Biochemical Technology Co., Ltd. (Shanghai, China). Reverse transcription reagents were purchased from Vazyme Biotech Co., Ltd. DSS salt colitis grade (36,000 to 50,000 Da) was purchased from MP Biomedicals (Santa Ana, CA, USA). The aspartate aminotransferase (AST) assay kit, alanine aminotransferase (ALT) assay kit, myeloperoxidase (MPO) assay kit, and urine fecal occult blood test kit were obtained from Nanjing Jiancheng Bioengineering Institute. The anti-F4/80, anti-Keratin5, anti-IL-6, and anti-TNF-α antibodies were obtained from Wuhan Servicebio Biotech Co., Ltd. The iNOS antibody was purchased from Proteintech. Other chemicals were obtained from Sinopharm Chemical Reagent Co., Ltd. All chemicals were of analytical grade and used directly without purification. Water used throughout all experiments was prepared using a Milli-Q water system.

### 2.2 Preparation of BiHANs

BiHANs nanoparticles were prepared via a mild one-step hydrothermal method as previously described with modification [36]. Here, 200 mg of HA (Sigma-Aldrich) was dissolved in 8.0 mL of ddH_2_O under magnetic stirring to obtain HA solution and pH of the solution was adjusted to 11 ± 0.2 using 0.1 M NaOH. 50 mg of Bi(NO_3_)_3_·5H_2_O was added to 1.0 mL HNO_3_ solution (2M) to be totally dissolved and then dropped into above humic acids solution under vigorous stirring at room temperature for 30 min. Subsequently, 30 mg FeCl_3_·6H_2_O was added to stir for 12 h at room temperature. After the reaction, the resulting mixture was centrifuged, and the supernatant was discarded. The precipitation was further purified by ddH_2_O dialysis for 24 h to remove excess reactants. Finally, BiHANs were obtained by vacuum freeze-drying overnight for further usage.

### 2.3 Characterization of BiHANs

The morphology and size of the nanoparticles were characterized by transmission electron microscopy (TEM, JEM-F200, Japan). Particle size distribution and zeta potential were determined using dynamic light scattering (DLS, ZS90 Zeta Sizer, Malvern, UK). The elemental distribution of the nanoparticles was assessed via high-resolution transmission electron microscopy (HR-TEM, HITACHI HT7800, Japan) coupled with energy-dispersive X-ray spectroscopy (EDS, JED-2300T, Japan). Ultraviolet–visible (UV–Vis) spectra were recorded using a UV–Vis spectrometer (Thermo Fisher, USA). Fourier-transform infrared (FTIR) spectra were obtained with a Fourier-transform infrared spectrometer (Thermo Scientific Nicolet iS20, USA). In vivo nanoparticle distribution was investigated using inductively coupled plasma mass spectrometry (ICP-MS, Agilent, USA). The binding energy of bismuth was measured by X-ray photoelectron spectroscopy (XPS, Thermo Fisher Nexsa, USA), and the crystalline structure of the nanoparticles was analyzed using X-ray diffraction (XRD, Panalytical X'Pert3 Powder, Netherlands).

### 2.4 Preparation of Cy5.5 labeled BiHANs (Cy5.5@ BiHANs)

The Cy5.5@BiHANs were synthesized via an acylation reaction between the amine group of Cy5.5 and the carboxyl group of HA. In a typical procedure, 8 mg of BiHANs was dissolved in 3 mL of MES buffer solution. Subsequently, 22 mg of Sulfo-NHS and 8 mg of EDC were added to the solution. After stirring for 30 min at room temperature, 0.5 mg of Cy5.5-NH_2_ was introduced into the reaction mixture, which was then magnetically stirred in the dark for 2 h. The mixture was purified by dialysis against distilled water to remove any unreacted Cy5.5-NH_2_. After dialysis, the products were lyophilized to obtain the final product for subsequent use.

### 2.5 Stability of BiHANs under different physiological conditions

BiHANs were initially dispersed in phosphate-buffered saline (PBS, 25 mM, pH 7.4), Dulbecco's Modified Eagle Medium (DMEM), gastric fluid, and intestinal fluid at a concentration of 200 μg/mL. The solutions were incubated at 25 °C. After each co-incubation period, photographs of BiHANs in the solutions were taken to observe their dispersion and stability after 7 days. Following centrifugation and freeze-drying, the BiHANs samples, which underwent different treatments, were analyzed using dynamic light scattering (DLS) and zeta potential measurements.

### 2.6 Scavenging ability against DPPH· of BiHANs

The ability of BiHANs to scavenge DPPH free radicals was evaluated using the DPPH assay (TargetMol). In this assay, BiHANs at different concentrations were added to PBS containing DPPH (100 μM), with anhydrous ethanol serving as the control. After incubation in the dark at room temperature for 30 min, absorbance at 517 nm was measured. The scavenging percentage was calculated using the following formula:

Scavenging percentage (%) = [(A_0_ - A)/A_0_] × 100, where A_0_ represents the absorbance of the control, and A is the absorbance of the mixture after the reaction. All measurements were performed in triplicate.

### 2.7 ROS-scavenging activity of BiHANs

First, 50 μM of DCFH-DA in PBS was incubated with 1 mM H_2_O_2_ at 37 °C. Different concentrations of BiHANs nanoparticles (6.25, 12.5, 25, 50, and 100 μg/mL) were then added to the solution and incubated in the dark for 4 h. Fluorescence signals were measured using a multi-mode microplate reader with 490 nm excitation and 520 nm emission. The ROS-scavenging rate was calculated using the following formula.

### 2.8 Cell culture

The mouse fibroblast cell line (L-929), human colorectal adenocarcinoma cell line (Caco-2), and mouse mononuclear macrophage leukemia cell line (RAW 264.7) were obtained from the Cell Bank of the Chinese Academy of Sciences (Shanghai). L-929 cells were cultured in MEM medium supplemented with 10 % FBS and 1 % PS (100 × ), without glutamine. RAW 264.7 cells were cultured in DMEM medium containing 10 % FBS and 1 % PS (100 × ). Caco-2 cells were cultured in MEM medium supplemented with 20 % FBS and 1 % PS (100 × ). All cell lines were incubated at 37 °C in a 5 % CO_2_ atmosphere.

### 2.9 *In vitro* cytotoxicity evaluation of BiHANs and Cy5.5@BiHANs

L-929 cells and RAW 264.7 cells were seeded in 96-well plates at a density of 10^4^ cells/well and allowed to adhere overnight. After cell adherence, BiHANs at varying concentrations (6.25 μg/mL, 12.5 μg/mL, 25 μg/mL, 50 μg/mL, 100 μg/mL, and 200 μg/mL) were added to the wells and cultured for an additional 24 h. Following incubation, the medium containing BiHANs was removed, and the cells were washed three times with PBS. The cells were then treated with MTT reagent for 4 h, after which 100 μL of DMSO was added to dissolve the formazan crystals. Absorbance was measured at 570 nm using a SpectraMax iD3 multifunctional enzyme reader. The same procedure was followed to evaluate the toxicity of Cy5.5@BiHA. Cell viability was calculated using the following equation:

Cell Viability (%) = (OD_treated_/OD_control_) × 100, where OD_control_ represents the absorbance in the absence of samples and OD_treated_ corresponds to the absorbance in the presence of samples.

### 2.10 Live/dead staining

The Calcein AM/PI cell viability assay was used to assess the cytotoxicity of BiHANs. Caco-2 cells were initially seeded in 6-well plates at a density of 5 × 10^5^ cells/well. After cell adhesion, BiHANs at varying concentrations were added to the wells and cultured for an additional 24 h. Following incubation, the medium containing BiHANs was replaced with Calcein AM/PI working solution, and the cells were incubated in the dark for 30 min. After rinsing with cold PBS, fluorescence signals from stained Caco-2 cells were analyzed and captured using a confocal microscope.

### 2.11 Cellular uptake

RAW 264.7 cells were initially seeded in 24-well plates at a density of 5 × 10^4^ cells/well. After cell adherence, Cy5.5@BiHANs at varying concentrations (10 μg/mL, 20 μg/mL, and 40 μg/mL) were added to the wells. The cells were then incubated at 37 °C for 24 h. After incubation, the cells were rinsed with PBS, fixed with 4 % paraformaldehyde, stained with DAPI, sealed with neutral resin, and imaged using a laser confocal microscope (Nikon, Japan).

### 2.12 The protective ability of BiHANs against oxidative stress damage

The CCK-8 assay kit was used to evaluate the ability of BiHANs to protect cells from H_2_O_2_-induced oxidative stress. Caco-2 cells were initially seeded in 96-well plates at a density of 1.3 × 10^4^ cells/well and incubated with H_2_O_2_ (800 μM) in combination with BiHANs at varying concentrations for 24 and 48 h. After incubation, the medium was removed, and 100 μL of fresh serum-free medium was added to each well. Cell viability was then assessed using the CCK-8 assay kit according to the manufacturer's instructions.

### 2.13 Intracellular ROS-scavenging efficiency on RAW 264.7 cells stimulated by LPS

RAW 264.7 cells were seeded in 24-well plates with cell slides at a density of 5 × 10^4^ cells/well and incubated overnight. The following day, the cells were cultured with medium containing 10 μg/mL of BiHANs in the presence of LPS (10 μg/mL) for 6 h. After incubation, the cells were stained with 50 μM DCFH-DA and incubated for 30 min at 37 °C. Following three washes with PBS, the cells were fixed with 4 % paraformaldehyde, stained with DAPI, and sealed with neutral resin. The cells were then observed qualitatively using a confocal microscopy imaging system (Nikon, Japan). Imaging results were quantitatively analyzed using ImageJ.

### 2.14 Mitochondrial function assessment in RAW 264.7 cells

The JC-1 kit (Aladdin) was used to assess changes in mitochondrial membrane potential following different treatments. RAW 264.7 cells were seeded in 24-well plates with cell slides at a density of 5 × 10^4^ cells/well and incubated overnight. The next day, the cells were cultured in medium containing 10 μg/mL of BiHANs and LPS (10 μg/mL) for 4 h. After washing with PBS three times, 1 mL of JC-1 staining solution was added to each well, and the plates were incubated at 37 °C for 30 min. Following three washes with staining buffer, the cells were fixed with 4 % paraformaldehyde at room temperature for 30 min, followed by DAPI staining for 15 min. Finally, the plates were sealed with neutral resin. Fluorescence images were acquired using a confocal laser scanning microscope (CLSM) with 488 nm excitation and 525 nm emission, and the imaging results were quantitatively analyzed using ImageJ.

### 2.15 In vitro anti-inflammatory assay

RAW 264.7 cells were initially seeded into 24-well plates at a density of 8 × 10^4^ cells/well and incubated overnight. The culture medium was then replaced, and the cells were treated with BiHANs at different concentrations for 24 h, followed by LPS treatment (10 μg/mL) for 4 h. Total RNA was extracted using the Invitrogen Trizol method, and 1000 ng of RNA was used for reverse transcription according to the Nanjing Novozymes kit (NO: R223-01) instructions. The mRNA levels of *TNF-α*, *IL-6*, and *IL-1β* were quantified using real-time PCR (CFX96 Real-Time PCR System) following the Nanjing Novozymes kit (NO: Q311-02) instructions. GAPDH was used as the reference gene. Primer sequences are provided in Table S1.

### 2.16 Animals and ethics statement

SPF C57BL/6J mice (male, 6–8 weeks old, weighing 22–25 g) were purchased from Charles River (Beijing). The mice were randomly assigned to groups of six per cage and allowed to acclimate for one week prior to the study. All animal experiments were conducted in accordance with the guidelines set forth by the Wenzhou Medical University Animal Care and Use Committee (approval number: xmsq2024-0205).

### 2.17 Hemolysis assay

Whole blood was obtained from healthy C57BL/6J mice using the eyeball blood sampling method. Fresh blood was collected into an anticoagulant tube containing EDTA and centrifuged at 3000 rpm for 15 min to isolate blood cells for the evaluation of the hemolytic rate of BiHANs. Subsequently, 1 mL of BiHANs at various concentrations (25 μg/mL, 50 μg/mL, 75 μg/mL, 100 μg/mL, 200 μg/mL), 1 mL of ddH_2_O, and 1 mL of PBS solution were each mixed with 20 μL of the obtained blood cells. All samples were incubated at 37 °C for 4 h. After centrifugation at 3000 rpm for 15 min, hemolysis was captured using a digital camera, and the absorbance of the supernatants at 542 nm was measured using an enzyme reader. The hemolytic rate (%) was calculated using the formula: Hemolytic rate (%) = [(A_sample_-A_PBS_)/(A_ddH2O_-A_PBS_)] × 100 %.

### 2.18 *In vitro* and *in vivo* CT targeted imaging

To assess the relationship between CT signals and BiHANs concentration, BiHANs at various concentrations were placed in 200 μL centrifuge tubes for *in vitro* CT signal detection, with an equivalent concentration of Ioversol used as the control group.

For *in vivo* CT imaging of BiHANs, we conducted animal experiments using a DSS-induced IBD murine model. Mice were administered 2 % (w/v) DSS for 7 consecutive days to induce colitis inflammation, followed by normal water until the end of the experiment. Healthy mice were provided with normal water only. On the 7th day after DSS administration, treatment was initiated for *in vivo* CT imaging. The experimental groups included: NC (healthy mice + gavage with H_2_O), NC + BiHANs (healthy mice + gavage with BiHANs), NC + Ioversol (healthy mice + gavage with Ioversol), DSS (IBD mice + gavage with H_2_O), DSS + BiHANs (IBD mice + gavage with BiHANs), and DSS + Ioversol (IBD mice + gavage with Ioversol).

Prior to CT scanning, all mice were anesthetized via intraperitoneal injection of pentobarbital sodium solution (1 wt%). At 24, 48, and 96 h post-administration, the mice were anesthetized with isoflurane and imaged using a Bruker Skyscan 1276 CT imaging system.

For *ex vivo* CT imaging, mice were administered BiHANs via oral gavage. Mice were sacrificed at 48 h post-administration. Prior to CT scanning, all mice were fasted for 12 h. Mice were anesthetized with 1 % sodium pentobarbital via intraperitoneal injection. Once the mice were fully anesthetized, they were dissected, and all organ tissues, including the heart, lungs, liver, stomach, spleen, kidneys, and intestinal segments, were collected for imaging using the Bruker Skyscan 1276 CT imaging system. The samples were then diluted with 2 M HNO_3_, and the amounts of Bi were measured using ICP-MS.

### 2.19 *In vivo* and *ex vivo* fluorescence imaging of Cy5.5-labeled BiHANs (Cy5.5@BiHANs) in mice

To investigate the ability of BiHANs to target the inflamed colon site and their biodistribution, mice were administered 2 % (w/v) DSS for 7 consecutive days to induce colitis inflammation, followed by normal water until the end of the experiment. Healthy control mice were provided with normal water only. The experimental groups were as follows: Cy5.5 (normal mice + gavage with Cy5.5), NC + Cy5.5@BiHANs (normal mice + gavage with Cy5.5@BiHANs), and DSS + Cy5.5@BiHANs (IBD mice + gavage with Cy5.5@BiHANs). At various time points (6 h, 12 h, 24 h, 48 h, 72 h, and 96 h post-administration), mice were anesthetized using isoflurane. Once the mice were fully anesthetized, they were placed in the IVIS Lumina Series Imaging System for fluorescence imaging.

For *ex vivo* imaging, mice were administered fluorescent Cy5.5@BiHANs via oral gavage. Mice were sacrificed at 6 time points, including pre-treatment, 6 h, 12 h, 24 h, 48 h, and 96 h post-administration. Mice that did not receive fluorescent Cy5.5@BiHANs were designated as the pre-treatment group. Organs, including the heart, kidneys, lungs, spleen, liver, small intestine, and colon, were harvested at the designated time points. Fluorescence imaging of the collected organ tissues was performed using the ChemiDoc MP Imaging System with a Cy5.5 filter channel and an exposure time of 0.5 s.

### 2.20 Colon localization of BiHANs

To further confirm the localization of BiHANs in the inflamed colon, mice were first fed with 2 % (w/v) DSS for 7 consecutive days to induce colitis inflammation, followed by normal water until the end of the experiment. Mice were sacrificed 24 h post-administration of BiHANs (24 mg/kg). Colon tissue was collected, rinsed with PBS, and immediately fixed in 2.5 % glutaraldehyde at 4 °C for 1 day. The sample was washed three times with PBS (pH 7.4) for 15 min each, followed by fixation with 1 % OsO_4_ in PBS for 1 h at room temperature, away from light. After removal of OsO_4_ and washing three times with PBS (15 min each), the specimen was sequentially immersed in acetone solutions of 30 %, 50 %, 70 %, 80 %, and 95 %, with gentle shaking for 15 min at each concentration. The sample was then dehydrated at room temperature, followed by resin infiltration, embedding, and polymerization. The resin blocks were cut into 70–90 nm ultra-thin sections using an ultra-thin microtome and placed onto copper grids. The samples were stained with a saturated uranyl acetate solution for 8–15 min, followed by lead citrate staining for 5–10 min. After rinsing with 70 % ethanol and ultrapure water (three times each), the copper grids were placed on a grid holder and dried overnight at room temperature. The morphology of the samples was observed under TEM (HITACHI HT7800/HT77000), and images were captured and analyzed.

### 2.21 *In vivo* safety assessment of BiHANs

Healthy mice were randomly assigned to two groups: the control group and the BiHANs group. Mice in the BiHANs group were orally administered 150 μL of BiHANs (24 mg/kg) every other day, while the control group received 150 μL of H_2_O. The mice were monitored for 48 days to assess *in vivo* biosafety. Body weight was recorded every other day. At the indicated time points, mice were euthanized, and blood samples were collected for the measurement of alanine aminotransferase (ALT) and aspartate aminotransferase (AST) levels. Major organs, including the heart, liver, spleen, lungs, kidneys, were harvested, fixed in 10 % paraformaldehyde, processed into paraffin, sectioned to ∼4 μm, and stained with hematoxylin and eosin (H&E). To evaluate nanoparticle accumulation, bismuth content in serum, small intestine, and colon tissues was determined using inductively coupled plasma mass spectrometry (ICP-MS).

### 2.22 Therapeutic efficacy of the nanoparticles in DSS-induced colitis

Chronic colitis model: Chronic Colitis Model: 36 male mice of 8-week-old age were randomly divided into six groups:

Group 1: Negative control (NC, healthy mice gavaged with H_2_O)

Group 2: HA (healthy mice gavaged with 24 mg/kg of HA)

Group 3: BiHANs (healthy mice gavaged with 24 mg/kg of BiHANs)

Group 4: DSS (IBD model mice gavaged with H_2_O)

Group 5: DSS + HA (IBD model mice gavaged with 24 mg/kg of HA)

Group 6: DSS + BiHANs (IBD model mice gavaged with 24 mg/kg of BiHANs)

Mice underwent three cycles of DSS administration. Each cycle consisted of 7 days of 2 % (w/v) DSS (MW 36–50 kDa) in drinking water, followed by 9 days of normal water. From day 2 of each cycle, mice received oral administration of either 24 mg/kg of HA, 24 mg/kg of BiHANs, or water every other day, with a total of 24 administrations during the experiment. Throughout the experiment, changes in body weight, fecal consistency, and colon bleeding were monitored every two days. Fecal samples were collected to detect occult blood, and the Disease Activity Index (DAI) was calculated based on weight loss, fecal consistency, and fecal blood volume. On the last day of the experiment, fecal samples were collected for 16S rRNA analysis of the intestinal microbiota (Wekemo). At the end of the experiment, mice were euthanized, spleen weight was recorded, and the colon was collected for measurement of colon length, histological evaluation, cytokine determination, MPO content detection, and immunohistochemical analysis.

The major organs (heart, liver, spleen, lungs, kidneys, stomach, small intestine, and colon) were harvested, and Bi distribution was analyzed using ICP-MS to assess the biodistribution of Bi in the tissues. This provided a detailed toxicological assessment of BiHANs therapy. Notably, mice from Groups 1 and 3 were also included in the *in vivo* safety assessment of BiHANs described earlier.

Acute Colitis Model:

To establish an acute colitis model, 24 male mice of 8-week-old age were randomly divided into four groups:

Group 1: Negative control (NC, healthy mice gavaged with H_2_O)

Group 2: DSS (IBD model mice gavaged with H_2_O)

Group 3: DSS + Bismuth Tratrate (BT) (IBD model mice gavaged with 24 mg/kg of Bismuth Tratrate)

Group 4: DSS + BiHANs (IBD model mice gavaged with 24 mg/kg of BiHANs)

Mice were given 3 % DSS in drinking water for 7 days to induce the acute colitis model, followed by 1 % DSS in drinking water. Healthy control mice received normal water only. Starting from day 2, mice were orally administered 24 mg/kg of Bismuth Tratrate (BT), 24 mg/kg of BiHANs, or water every other day, with a total of 8 administrations throughout the experiment. Changes in body weight, stool viscosity, hematochezia, physical condition, and activity were monitored using the same protocol as in the chronic colitis model. On the final day of the experiment, mice were euthanized, and the entire colon and spleen were harvested. Spleen weight was recorded, and colons were collected for colon length measurement, histological evaluation, MPO content detection, and immunofluorescence staining.

### 2.23 Measurement of MPO activity

The colon tissue was accurately weighed and then homogenized with a medium at a weight-to-volume ratio of 1:19. The sample was processed using a high-speed, low-temperature tissue homogenizer to ensure thorough mixing. Following homogenization, the sample was incubated at 60 °C in a water bath for 10 min. The absorbance of each well was measured at 460 nm immediately after incubation. Myeloperoxidase (MPO) activity was calculated using the following formula: MPO activity (U/g) = (A_experiment_-A _control_)/(11.3 × W), where A_experiment_ is the absorbance of the experimental group, A _control_ is the absorbance of the control group, W is the tissue sample sampling amount (g), and W = homogenate concentration (5 %) × sampling volume (0.18 mL).

### 2.24 Immunohistochemistry staining of IL-6 and TNF-α

Colon tissue was initially fixed in 4 % paraformaldehyde solution, followed by paraffin embedding and sectioning. The thin sections were then dewaxed, hydrated, and subjected to antigen retrieval before blocking nonspecific binding. The sections were incubated sequentially with primary and secondary antibodies and then stained with 3,3′-diaminobenzidine (DAB). Once the tissue sections showed a slight yellow tint, they were promptly rinsed with tap water to halt the staining process. Subsequently, the sections were stained with DAPI for nuclear visualization at room temperature in the dark for 15 min. The slides were then immersed in hematoxylin solution for 2 min, followed by a 10-min wash with tap water. Next, the slides were sequentially treated with 75 % ethanol for 5 min, 85 % ethanol for 5 min, absolute ethanol I for 5 min, and absolute ethanol II for an additional 5 min, followed by dehydration. After natural air-drying, the sections were mounted with neutral resin and visualized under an immunohistochemical microscope.

### 2.25 Immunohistochemistry staining of Keratin5 and F4/80

The distal colon sections were fixed in paraformaldehyde and subsequently dehydrated in ethanol. The tissue was then embedded in paraffin and sectioned into 4 μm thick slices. Antigen retrieval was performed using a heat-induced epitope retrieval (HIER) method, followed by blocking with goat serum to prevent nonspecific binding. The tissue sections were incubated overnight at 4 °C with primary antibodies: anti-Keratin 5 and anti-F4/80, in a humidified chamber. Afterward, the sections were incubated at room temperature in darkness for 50 min with fluorophore-conjugated secondary antibodies: Alexa Fluor 488-labeled goat anti-rat IgG and Cy3-labeled goat anti-rat IgG. Nuclear staining was performed using DAPI. Finally, the images were captured using an optical microscope (ECLIPSE C1, Nikon, Japan).

### 2.26 Microbiome analysis

Fresh fecal pellets (6 per mouse) were collected for analysis. The samples were immediately labeled and placed in liquid nitrogen for subsequent intestinal microbiome analysis. The 16S rRNA sequencing of the microbiome was performed by Wekemo Tech Group Co., Ltd. (Shenzhen, China). The V3-V4 hypervariable region of the 16S rRNA gene (Sangon Biotech) was targeted in this study. Briefly, microbial genomic DNA was extracted using the E.Z.N.A. Mag-Bind Soil DNA Kit (OMEGA). DNA quality was assessed via electrophoresis on a 2 % agarose gel. The DNA concentration was measured using a Qubit 3.0 fluorometer (Thermo Fisher Scientific). The target region was amplified using primer pairs Nobar_341F (5′-CCTACGGGNGGCWGCAG-3′) and Nobar_805R (5′-GACTACHVGGGTATCTAATCC-3’). Sequence analysis was performed using Wekemo Bioincloud (https://www.bioincloud.tech). Pearson correlation analysis were performed with Origin 2024 software.

### 2.27 Colocalization assay of Cy5.5@BiHANs and mitochondria in RAW 264.7 cells

RAW 264.7 cells (5 × 10^4^) were seeded into 24-well culture plates, with coverslips placed in each well. After cell attachment, BiHANs or Cy5.5@BiHANs were added to the wells at a final concentration of 10 μg/mL and incubated for 24 h. The culture medium was then removed, and cells were stained with Mito-Tracker Green working solution, ensuring uniform distribution through gentle agitation. Cells were incubated at 37 °C for 30 min, after which the staining solution was aspirated, and cells were washed 2–3 times with PBS. Next, cells were fixed with a fixation solution and counterstained with DAPI to visualize nuclei. Finally, coverslips were mounted, and cells were observed under a fluorescence microscope.

### 2.28 Intracellular ROS-scavenging efficiency on RAW 264.7 cells stimulated by DSS

RAW 264.7 cells were seeded into 24-well plates containing cell slides at a density of 5 × 10^4^ cells per well and incubated overnight. The following day, the cells were cultured in medium containing 10 μg/mL of BiHANs and 2 mg/mL of DSS for 6 h. After incubation, the cells were stained with 50 μM DCFH-DA and incubated for an additional 30 min at 37 °C. The cells were then washed three times with PBS, fixed with 4 % paraformaldehyde, and counterstained with DAPI. Finally, the cells were mounted with neutral resin and observed under a confocal microscopy imaging system (Nikon, Japan) for qualitative analysis.

### 2.29 DSS-induced mitochondrial permeability transition pore (MPTP) changes in RAW 264.7 cells

A total of 5 × 10^4^ RAW 264.7 cells were seeded into a 24-well cell culture plate with coverslips. After cell adhesion, DSS (final concentration: 2 mg/mL) and BiHANs (10 μg/mL) were added, followed by incubation for 24 h. The culture medium was then aspirated, and the cells were washed twice with PBS. Subsequently, 250 μL of Calcein AM staining solution, fluorescence quenching working solution, or Ionomycin control was added, ensuring uniform distribution by gentle agitation. Cells were incubated at 37 °C in the dark for 30–45 min. After incubation, the staining solution was replaced with fresh pre-warmed (37 °C) culture medium, and the cells were further incubated in the dark at 37 °C for an additional 30 min to allow intracellular esterases to hydrolyze Calcein AM into the green fluorescent Calcein. The culture medium was then aspirated, and the cells were washed 2–3 times with PBS before fixation. DAPI staining was performed for nuclear visualization, followed by coverslip mounting and fluorescence microscopy observation.

### 2.30 Statistical analysis

All results were expressed as mean ± standard deviation of at least three independent experiments, and statistical analysis was performed using GraphPad Prism 8.0 (GraphPad Software, San Diego, CA). T-test was used to compare two independent groups of samples or one-way analysis of variance (ANOVA) when multiple groups were compared. The test standard was *p* value less than 0.05 for statistically significant differences, ns for *p* > 0.05, ∗ for *p* < 0.05, ∗∗ for *p* < 0.01, ∗∗∗ for *p* < 0.001, and ∗∗∗∗ for *p* < 0.0001.

## 3 Result

### 3.1 Synthesis and characterization of BiHANs

BiHANs were synthesized via a well-defined chelation-driven self-assembly approach, following a previously established protocol [36]. In this process, HA was mixed with a solution of bismuth nitrate pentahydrate (Bi(NO_3_)_3_·5H_2_O), following a previously established protocol (Scheme 1A, Fig. S1). Detailed experimental procedures are provided in the experimental section. During synthesis, functional groups in HA, such as carboxyl, phenolic hydroxyl, carbonyl, and amino groups, coordinate with Bi^3+^ ions to form Bi–O active sites. A subsequent reduction step in an alkaline environment stabilizes the nanostructures by interacting with Bi^3+^ binding sites on HA. The size of the BiHANs was found to be influenced by the Bi^3+^/HA ratio (Fig. S2). Among the synthesized BiHANs, those prepared at a Bi^3+^:HA mass ratio of 1:4 exhibited excellent dispersity and a small size, making them ideal for further characterization. The resulting BiHANs were purified through dialysis, lyophilization, and multiple washing steps to remove unreacted precursors and ensure high purity.

All components used in the synthesis are cost-effective and commercially available, rendering the process both economical and user-friendly. Notably, BiHANs can be utilized directly for bioimaging and administration without requiring surface modification, significantly enhancing their potential for clinical and commercial translation.

**Fig. 1:**
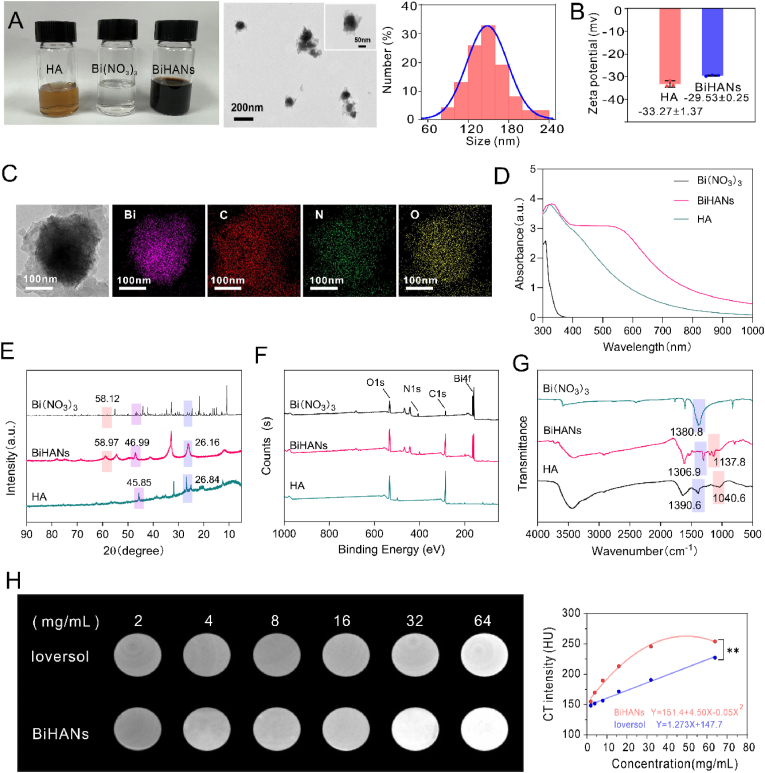
Synthesis and characterization of BiHANs. A) The left picture shows electron micrographs of HA, Bi(NO_3_)_3_, and the BiHANs solution. In the middle is the TEM image of the BiHANs, and on the right is the TEM particle size distribution statistical chart. B) Zeta potential of HA and BiHANs. C) EDS mapping of the Bi, C, N, and O elemental signals of the BiHANs. D) Absorption spectra of HA and BiHANs. E) XRD analysis of HA, Bi(NO_3_)_3_ and BiHANs. F) XPS survey spectra of HA, Bi(NO_3_)_3_ and BiHANs. G) FTIR analysis of HA, Bi(NO_3_)_3_ and BiHANs. H) CT image and value (HU) of BiHANs and Ioversol at different concentrations. Data were analyzed by paired *t*-test (H).

BiHANs were characterized using a range of analytical techniques. The synthesized BiHANs exhibited a black color and demonstrated excellent dispersibility in aqueous solution. Transmission electron microscopy (TEM) images revealed uniformly spherical particles with an average diameter of approximately 148 nm (Fig. 1A). Dynamic light scattering (DLS) measurements indicated an average hydrodynamic size of 172 nm (Fig. S3), which was slightly larger than the TEM-derived size due to nanoparticle hydration in solution. The zeta potential of HA was measured at −33.27 ± 1.37 mV, which increased to −29.53 ± 0.25 mV for BiHANs, confirming the successful chelation of Bi^3+^ ions (Fig. 1B). High-resolution TEM (HR-TEM) combined with energy-dispersive spectroscopy (EDS) mapping demonstrated a homogeneous distribution of Bi, C, N, and O within the BiHANs, with Bi accounting for 44.11 wt% of the composition (Fig. 1C, Fig. S4). Ultraviolet–visible (UV–vis) spectroscopy displayed a characteristic ligand-to-metal charge transfer band in the range of 400–550 nm, further confirming the coordination of Bi^3+^ with HA (Fig. 1D). X-ray diffraction (XRD) analysis revealed distinct peaks at 26.16°, 46.99°, and 58.97°, providing additional evidence for the binding of Bi^3+^ to HA (Fig. 1E). X-ray photoelectron spectroscopy (XPS) confirmed the presence of C, N, O, and Bi, with peaks at 159.49 eV (Bi4f) and 286.36 eV (O1s) corresponding to Bi–O and C–O coordination bonds, respectively (Fig. 1F, Fig. S5). Fourier transform infrared (FTIR) spectroscopy showed characteristic bands in the range of 1298–793 cm^−1^, indicative of Bi^3+^ coordination with the hydroxyl and carboxyl groups of HA (Fig. 1G).

The X-ray attenuation capability of BiHANs was evaluated using computed tomography (CT) imaging. BiHANs exhibited significantly higher sensitivity compared to the clinical contrast agent Ioversol at equivalent concentrations, attributed to the higher atomic number (Z = 83) and K-edge value (90.5 keV) of bismuth relative to iodine (Z = 53, K-edge = 33.2 keV) (Fig. 1H). These results underscore the potential of BiHANs as ultrasensitive CT contrast agents for *in vivo* imaging applications.

To evaluate their suitability for biomedical applications, the colloidal stability of BiHANs was assessed under physiological conditions. BiHANs demonstrated excellent stability over extended periods (e.g., 7 days) in physiological environments, as well as in simulated gastric fluid (SGF) and simulated intestinal fluid (SIF) (Fig. S6). These findings highlight the robustness of BiHANs for potential use in biomedical settings.

### 3.2 Antioxidant and anti-inflammatory activities of the BiHANs *in vitro*

**Fig. 2:**
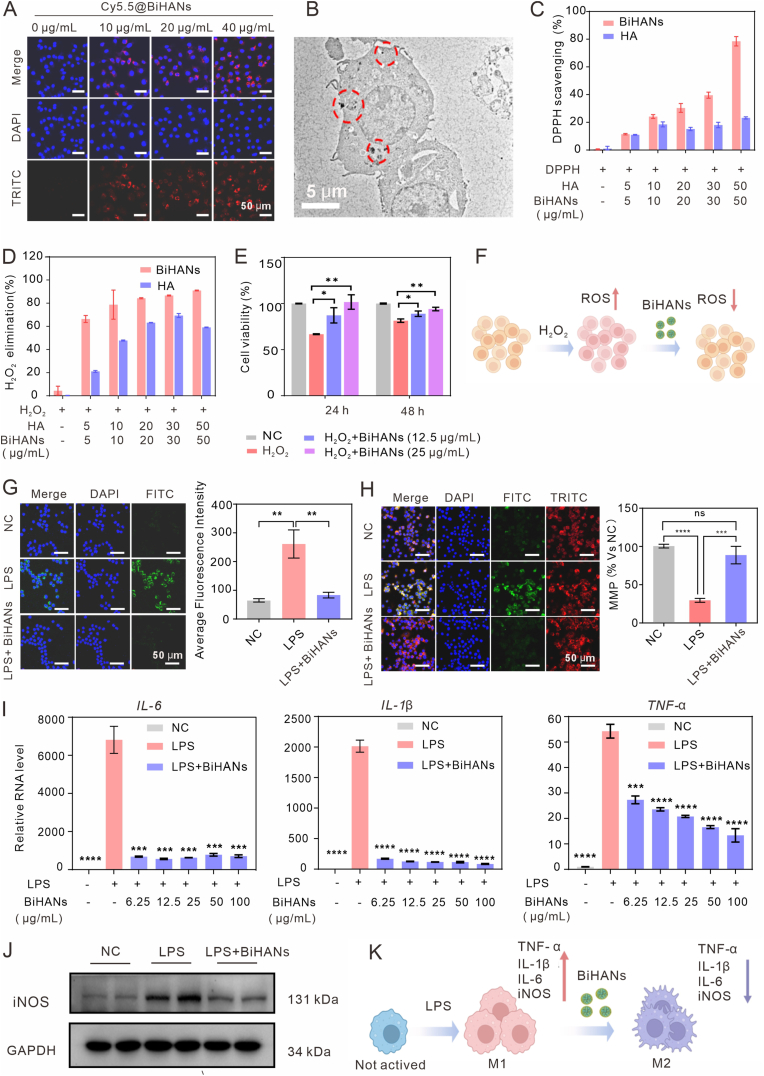
Cellular uptake, ROS scavenging ability, and anti-inflammatory effects of BiHANs. A, B) Cellular uptake of BiHANs in RAW 264.7 cells visualized via fluorescence microscopy and bio-TEM after 24 h of treatment. Scale bar: 50 μm. C) Quantitative analysis of the DPPH (100 μM) scavenging capacity of BiHANs at different concentrations. D) Quantitative analysis of the H_2_O_2_ (1 mM) scavenging capacity of BiHANs at different concentrations. E) Viability of H_2_O_2_-treated Caco-2 cells following various treatments. F) Schematic illustration of the ROS scavenging assay in H_2_O_2_-stimulated cells. G) Merged images of DCFH-DA (a ROS probe) staining (green) and DAPI staining (blue), along with fluorescence intensity analysis of RAW 264.7 cells under different treatment conditions. Scale bar: 50 μm. H) JC-1 fluorescence images (showing JC-1 monomers and aggregates) depicting mitochondrial membrane potential (Δψm) under different treatments, with corresponding fluorescence intensity quantification (mean ± SD, n = 3, *∗p < 0.05*, *∗∗p < 0.01*, *∗∗∗p < 0.001*, *∗∗∗∗p < 0.0001*). I) RT-qPCR analysis of *IL-1β*, *TNF-α*and *IL-*6 mRNA levels in RAW 264.7 cells after the indicated treatments. (J) Western blot analysis of iNOS and GAPDH protein expression in RAW 264.7 cells under different treatment conditions. (K) Schematic illustration of the LPS-induced RAW 264.7 cell polarization assay. Data were analyzed by Holm-Sidak's multiple comparisons test (E) and unpaired, two-sided Student's t-test (G–I). (For interpretation of the references to color in this figure legend, the reader is referred to the Web version of this article.)

To evaluate the cellular uptake efficiency of BiHANs, Cy5.5-labeled BiHANs were designed to track intracellular internalization using fluorescence imaging. Prior to uptake studies, CCK-8 assays confirmed the negligible cytotoxicity of both labeled and unlabeled BiHANs (Fig. S7). As shown in Fig. 2A, red fluorescence was observed in the cytoplasm of RAW 264.7 cells, with fluorescence intensity increasing in a concentration-dependent manner. The intracellular localization of BiHANs in RAW 264.7 cells after 24 h of treatment was further analyzed using bio-TEM. Fig. 2B demonstrates that BiHANs were effectively internalized and primarily localized within the cytoplasm of macrophages. Additionally, fluorescence imaging confirmed the uptake of BiHANs in Caco-2 cells (Fig. S8).

Given the critical role of antioxidant activity in IBD treatment, the free radical-scavenging capacity of BiHANs was evaluated using DPPH and H_2_O_2_ assays. As anticipated, BiHANs exhibited concentration-dependent ROS scavenging, outperforming free HA due to their metal–polyphenol nanostructure (Fig. 2C and D). To evaluate their stability in harsh physiological environments, the antioxidant activity of BiHANs was tested in simulated gastric fluid (SGF) and simulated intestinal fluid (SIF). Encouragingly, BiHANs maintained stable ROS-scavenging activity in both SGF and SIF (Fig. S9).

Next, the cellular antioxidative potential of BiHANs was investigated. CCK-8 assays revealed that Caco-2 cell viability decreased following stimulation with 1 mM H_2_O_2_. However, treatment with BiHANs restored cell viability, indicating protection against H_2_O_2_-induced oxidative damage (Fig. 2E and F). To further evaluate the anti-inflammatory effects of BiHANs, RAW 264.7 cells were treated with LPS. Intracellular ROS levels were assessed using DCFH-DA staining. As shown in Fig. 2G, LPS stimulation significantly increased green fluorescence, indicating elevated ROS production. Treatment with BiHANs markedly reduced green fluorescence intensity, demonstrating their effective intracellular ROS-scavenging capability.

Mitochondrial dysfunction is a hallmark of IBD [37]; therefore, the mitochondrial membrane potential (MMP) was assessed using the JC-1 fluorescence probe. In healthy mitochondria, JC-1 forms red fluorescent aggregates (J-aggregates), whereas ROS-induced damage disrupts this aggregation, leading to the accumulation of green fluorescent monomers (J-monomers). As shown in Fig. 2H, RAW 264.7 cells treated with LPS exhibited a significantly elevated J-monomer/aggregate ratio, indicating mitochondrial dysfunction. Notably, treatment with BiHANs restored red fluorescence by reversing LPS-induced mitochondrial damage, demonstrating their protective effect on the inner mitochondrial membrane.

Furthermore, the anti-inflammatory effects of BiHANs were investigated by quantifying cytokine expression via real-time quantitative PCR (qPCR). As shown in Fig. 2I, BiHANs significantly suppressed the expression of proinflammatory cytokines, including *TNF-α*, *IL-1β* and *IL-6* in RAW 264.7 cells (primer sequences are provided in Table S1). Western blot analysis was conducted to evaluate the expression of inducible nitric oxide synthase (iNOS). As shown in Fig. 2J, LPS stimulation markedly increased iNOS expression, indicating M1 polarization. Notably, treatment with BiHANs substantially reduced iNOS levels, as well as *TNF-α*, *IL-1β* and *IL-6* expression, indicating that BiHANs effectively suppressed LPS-induced M1 polarization in RAW 264.7 cells (Fig. 2K).

### 3.3 Biocompatibility evaluation of BiHANs

**Fig. 3:**
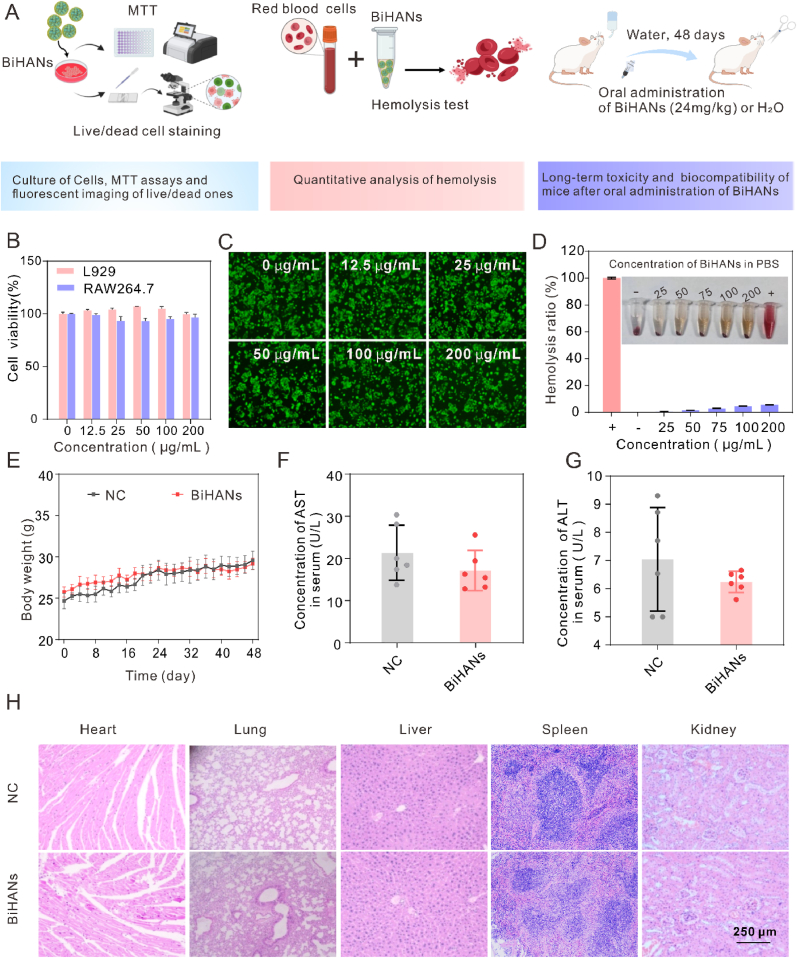
Biosafety Evaluation of BiHANs. A) Schematic illustration of the experimental protocol used to assess the toxicity of BiHANs. B) Viability of L-929 and RAW 264.7 cells following incubation with different concentrations of BiHANs. C) Live/dead staining images of Caco-2 cells after BiHANs treatment. D) Hemolysis analysis of BiHANs at varying concentrations. E) Body weight changes in each group over a 48-day period. F, G) Plasma levels of aspartate aminotransferase (AST) and alanine aminotransferase (ALT). Data are presented as mean ± SD (n = 6). H) Hematoxylin and eosin (H&E)-stained sections of major organs (heart, liver, kidney, lung, spleen) analyzed for systemic toxicity assessment.

The biocompatibility of BiHANs was comprehensively assessed through both *in vitro* and *in vivo* experiments. The experimental design is illustrated in Fig. 3A, with detailed procedures provided in the Supporting Information. Initially, the cytotoxicity of BiHANs toward L-929 and RAW 264.7 cells was evaluated using the standard MTT assay. As shown in Fig. 3B, no significant cytotoxicity was detected at any of the tested concentrations. Furthermore, BiHANs exhibited no adverse effects on Caco-2 cell viability, as confirmed by Live/Dead cell staining (Fig. 3C). Additionally, as shown in Fig. 3D and Table S2, the hemolysis rate remained below 6 % even at a BiHANs concentration of 200 μg/mL, highlighting their excellent hemocompatibility.

For *in vivo* evaluation, healthy mice were orally administered BiHANs every other day, and their body weight, blood biochemical indices, and histopathological changes were monitored (Fig. 3E–H). Throughout the 48-day observation period, no significant differences were observed between the BiHANs-treated and control groups, indicating the high biosafety of BiHANs following oral administration.

### 3.4 Intestinal localization and distribution *in vivo* of BiHANs

**Fig. 4:**
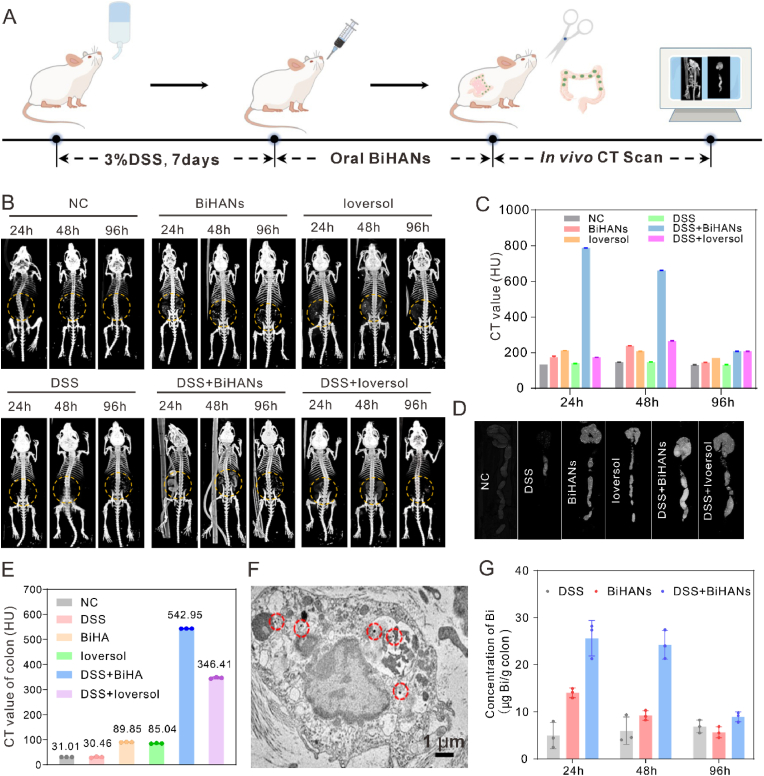
BiHANs target inflammatory sites and are visualized through CT imaging. A) Schematic illustration of BiHANs targeting inflammatory sites and facilitating specific CT imaging. B, C) Representative *in vivo* CT images of healthy and IBD mice after oral administration of BiHANs or Ioversol at various time points, along with quantitative analysis of the CT attenuation values in the intestinal tract across different groups at different times. D, E) Representative *ex vivo* images of healthy and IBD mice 48 h after treatment with BiHANs, and *ex vivo* quantitative analysis of CT attenuation values in the intestinal tract across different groups at 48 h post-treatment with BiHANs. F) Representative TEM images of the colon with high CT attenuation, showing BiHANs targeting the inflamed colonic epithelium (BiHANs indicated by the red arrow). G) ICP-MS measurement of Bi content in IBD mice after oral administration of BiHANs at different times. The data in panels C, E, and G (n = 3 per group) are presented as the mean ± S.D. (For interpretation of the references to color in this figure legend, the reader is referred to the Web version of this article.)

Due to the increasing use of CT imaging for evaluating abdominal symptoms in emergency settings, accurately visualizing and localizing IBD lesions has become essential for individualized clinical treatment. Emergency physicians, gastroenterologists, and general surgeons frequently encounter this challenge. After validating the CT imaging capabilities of BiHANs *in vitro* in previous studies, we next investigated their *in vivo* CT imaging potential. An IBD mouse model was established by administering 3 % (w/v) dextran sulfate sodium (DSS) in drinking water for seven consecutive days (Fig. 4A), with detailed experimental procedures provided in the Supporting Information. We then tracked the distribution of BiHANs in the intestine by CT imaging following oral administration of either BiHANs or Ioversol to healthy and IBD model mice. As shown in Fig. 4B and C, BiHANs accumulated at the IBD lesions 24 h post-administration, with CT signals persisting for up to 96 h. Furthermore, the CT signal intensity in the BiHANs + DSS group was significantly stronger than in other groups at 24, 48, and 96 h post-administration. In contrast, the colon CT intensity in the Ioversol + DSS group remained nearly identical to that in the Ioversol + healthy group at all time points, indicating that Ioversol does not specifically target IBD lesions when administered orally. Mice were euthanized 24 h after oral administration of BiHANs or Ioversol, and their colons were isolated for *ex vivo* CT imaging. *Ex vivo* CT imaging demonstrated significantly enhanced BiHANs accumulation in DSS-induced inflammatory lesions (6-fold vs. healthy controls; Fig. 4D and E), with spatial correlation to severe mucosal ulcerations identified by H&E staining (red arrows, Fig. S11). Healthy colon tissues exhibited negligible retention, confirming inflammation-specific targeting. Additionally, bio-TEM analysis confirmed the presence of BiHANs in the colon tissues of IBD-treated mice (Fig. 4F).

To further investigate the biodistribution and metabolism of BiHANs *in vivo*, Cy5.5-labeled BiHANs (Cy5.5@BiHANs) were administered to both healthy and DSS-induced IBD mice (Fig. S10A). Time-dependent fluorescence imaging was conducted using an IVIS imaging system. As shown in Fig. S10B and S10C, oral administration of Cy5.5@BiHANs resulted in significant accumulation in the inflamed colonic tissues, with minimal distribution in other major organs, which was consistent with the CT imaging results. Notably, the fluorescence signal from the Cy5.5 group was the weakest at 6-and 12-h post-administration due to rapid metabolism and excretion. In contrast, in IBD mice, Cy5.5@BiHANs exhibited strong gastrointestinal retention, with fluorescence signals detectable up to 48 h. Healthy mice showed consistently weaker fluorescence at all time points compared to the Cy5.5@BiHANs + DSS group, with the fluorescence signal progressively diminishing over time, and complete metabolism and excretion occurring within 48 h. To further assess biodistribution, *ex vivo* fluorescence imaging was performed after euthanasia. As shown in Fig. S10D, fluorescence intensity in the serum, heart, liver, lungs, spleen, kidneys, and stomach of healthy mice remained weak throughout the observation period. However, fluorescence signals were notably stronger in the small intestine and colon at 24 h, with no detectable signals in any organ by 48 h. Interestingly, in IBD mice, Cy5.5@BiHANs persisted for up to 48 h, predominantly accumulating in the colon.

Additionally, the bismuth (Bi) content in the colons of IBD mice was quantified using inductively coupled plasma mass spectrometry (ICP-MS). The Bi content in IBD mouse colons was approximately 1.8- and 2.7-fold higher than in healthy mice at 24-and 48-h post-administration, respectively, while remaining extremely low in other tissues. These findings strongly support the *in vivo* CT and fluorescence imaging results (Fig. 4G). Taken together, these results demonstrate that BiHANs can effectively and durably target inflamed colonic tissues and may be employed to assist in the diagnosis of IBD via CT imaging.

### 3.5 Therapeutic efficacy of BiHANs in DSS-induced chronic colitis

**Fig. 5:**
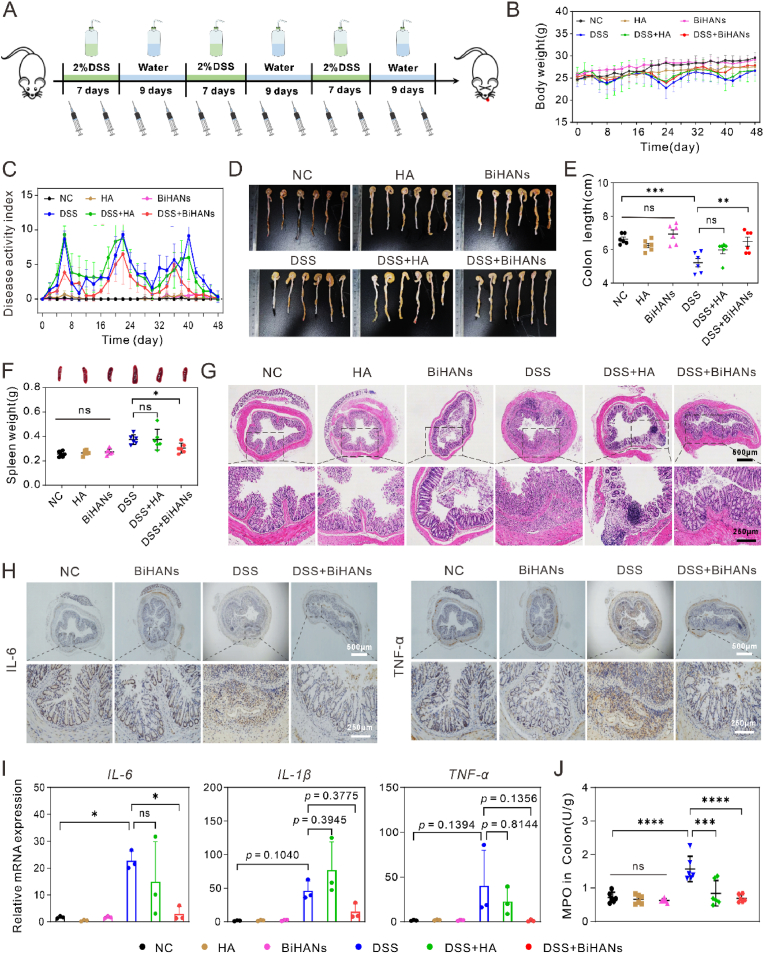
Therapeutic effects of BiHANs on DSS-induced chronic colitis. A) Schematic illustration of the experimental protocol for treating DSS-induced colitis with BiHANs. B, C) Changes in body weight and DAI in different treatment groups throughout the experimental period (n = 6). D, E) Images of colons and corresponding colon lengths from different treatment groups (n = 6). F) Digital photographs and changes in spleen weights harvested from the different treatment groups (n = 6). G) H&E-stained images of colonic sections from different groups. H) Representative immunohistochemical staining images of colonic sections from mice on the day of sacrifice. I) RT-qPCR analysis of mRNA levels of proinflammatory factors *IL-6*, *TNF-α* and *IL-1β* in colon homogenates (n = 3). J) MPO activity in colons from different treatment groups (n = 6). The data are presented as the mean ± S.D. *∗p* < 0.05, *∗∗p* < 0.01, *∗∗∗p* < 0.001; ns indicates no statistical difference. Data were analyzed by one-way ANOVA with Tukey's multiple comparisons test (E, I-J) or unpaired, two-sided Student's t-test (F).

Building on the robust antioxidant and anti-inflammatory properties of BiHANs demonstrated *in vitro*, we further evaluated their therapeutic potential in a chronic colitis mouse model. The model was induced by administering three cycles of 2 % dextran sulfate sodium (DSS), each lasting seven days, interspersed with nine-day water intervals. The schematic representation of the experimental design is shown in Fig. 5A. As illustrated in Fig. 5B–F, DSS-induced colitis resulted in significant weight loss, elevated disease activity index (DAI) scores (scoring criteria detailed in Table S3), shortened colon length, and spleen enlargement compared to the NC, HA, and BiHANs groups. In contrast, treatment with either HA or BiHANs in IBD mice mitigated these pathological changes, as evidenced by reduced weight loss, lower DAI scores, longer colon lengths, and reduced spleen enlargement compared to the DSS group. Notably, the DSS + BiHANs group showed superior outcomes, including greater body weight recovery, lower DAI scores, longer colon lengths, and reduced spleen enlargement, compared to the DSS + HA group.

Histopathological analysis via hematoxylin and eosin (H&E) staining revealed severe mucosal destruction, crypt damage, and extensive inflammatory cell infiltration in the DSS group (Fig. 5G). While the DSS + HA group exhibited mild structural improvements, significant inflammatory cell accumulation persisted. In contrast, the DSS + BiHANs group demonstrated better-preserved colonic architecture, with restored epithelial cells, reduced crypt damage, and less inflammatory cell infiltration.

Immunohistochemical staining of colonic tissues indicated elevated levels of the proinflammatory cytokines IL-6 and TNF-α in the DSS group, which were significantly attenuated following BiHANs treatment (Fig. 5H). Further validation through RT-qPCR and enzyme-linked immunosorbent assay (ELISA) confirmed that BiHANs effectively reduced levels of proinflammatory factors and reactive oxygen species (ROS), including *IL-6, IL-1β, TNF-α* and myeloperoxidase (MPO) (Fig. 5I and J). Importantly, BiHANs treatment did not induce detectable systemic toxicity or bismuth leakage in major organs (Fig. S12–S13).

In summary, these findings demonstrate that BiHANs effectively and safely alleviate chronic colitis. Notably, BiHANs exhibited significantly greater therapeutic efficacy than HA at an equivalent dose, likely due to their ability to form polyphenol-metal nanoassemblies, which enhance nanoparticle uptake and targeted therapeutic delivery.

### 3.6 Comparative efficacy of BiHANs and bismuth tartrate (BT) in DSS-induced acute colitis

**Fig. 6:**
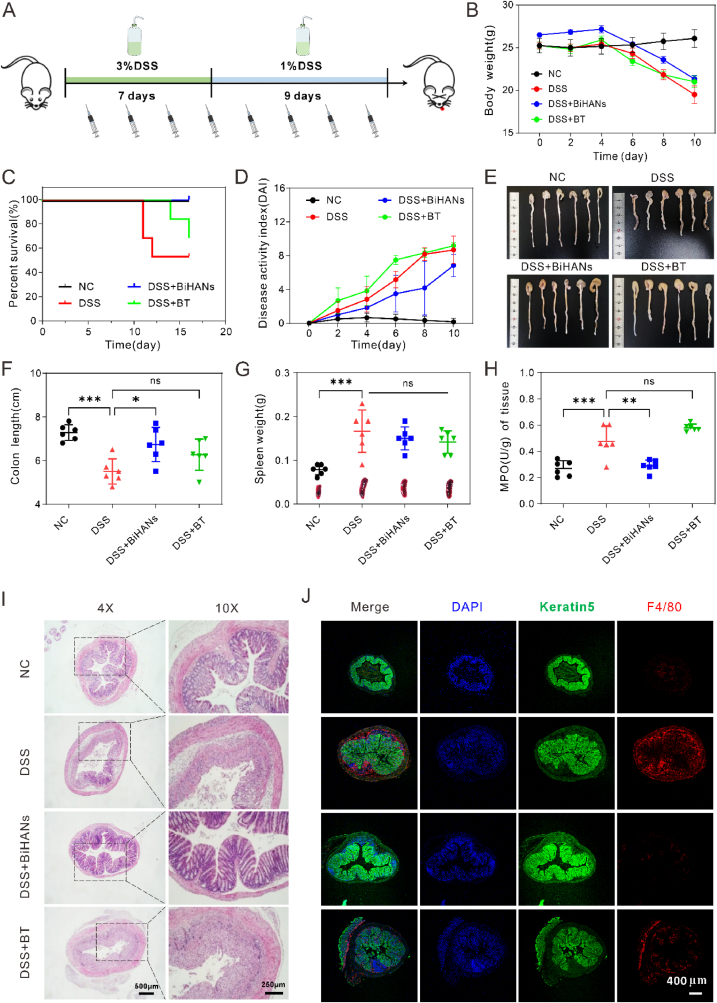
Comparative therapeutic effect of BiHANs and BT on DSS-induced acute colitis. A) Schematic diagram illustrating the colitis model preparation and treatment protocol. B) Body weights of mice in different groups throughout the experimental period (n = 6). C) Survival rates of mice following the indicated treatments over the entire experimental period. D) Disease Activity Index (DAI) scores for mice in each group throughout the experimental period (n = 6). E, F) Colon lengths and corresponding images of colons from different groups. G) Photographs and spleen weights of mice in different groups (n = 6). H) MPO activity in the colons of mice (n = 6). I) Hematoxylin and eosin (H&E) staining of colonic sections from mice (n = 6). J) Representative immunofluorescence images of F4/80 in colon tissue. Data are presented as the mean ± S.D. from a representative experiment (n = 6 biologically independent animals per group). Data were analyzed by one-way ANOVA with Tukey's multiple comparisons test (F–H). *∗p* < 0.05, *∗∗p* < 0.01, *∗∗∗p* < 0.001, *∗∗∗∗p* < 0.0001, and ns indicates no statistical difference.

To further explore the therapeutic potential of BiHANs, their efficacy in treating acute colitis was evaluated and compared with that of colloidal bismuth tartrate (BT), a clinically established treatment for diarrhea and peptic ulcer disease [38]. Acute ulcerative colitis was induced in mice by administering 3 % dextran sulfate sodium (DSS) for seven days, followed by 1 % DSS for nine days (Fig. 6A). Body weight was monitored every two days throughout the experimental period.

Results showed that body weight remained stable in the normal control (NC) group, while mice in the DSS + BT group exhibited weight loss similar to the DSS group. In contrast, weight loss in the DSS + BiHANs group was significantly mitigated compared to the DSS + BT group (Fig. 6B). Survival rates were 50.0 % in the DSS group and 66.7 % in the DSS + BT group, whereas all mice in the DSS + BiHANs group survived the experimental period, demonstrating a significant improvement in survival outcomes (Fig. 6C). The disease activity index (DAI) was significantly elevated in the DSS group compared to all other groups (Fig. 6D). While the DAI in the DSS + BT group was lower than in the DSS group, it remained elevated.

Colon length, a key indicator of colitis severity, was significantly reduced in the DSS group compared to the NC group (Fig. 6E and F). Although the DSS + BT group showed partial recovery, colon length did not return to baseline levels. In contrast, colon length in the DSS + BiHANs group was significantly restored to levels comparable to the control group, emphasizing the robust protective effect of BiHANs against DSS-induced colonic shortening.

To assess immune alterations, spleen weight was measured. As shown in Fig. 6G, spleen enlargement was evident in the DSS-induced colitis model, with no significant difference between the DSS and DSS + BT groups. However, spleen weight in the DSS + BiHANs group remained close to normal levels. MPO activity, a marker of neutrophil infiltration and inflammation, was also evaluated. MPO levels were significantly elevated in the DSS group, with no notable difference between the DSS and DSS + BT groups. In contrast, MPO activity in the DSS + BiHANs group was significantly reduced, returning to normal levels (Fig. 6H). These findings collectively demonstrate that oral administration of BiHANs effectively alleviated IBD symptoms, as evidenced by reduced body weight loss, restored colon length, normalized spleen weight, lower DAI scores, and attenuated MPO activity. Notably, BT failed to exert significant therapeutic effects on these parameters at the same dosage as BiHANs.

Histological analysis further supported these results. H&E staining of colon sections revealed severe mucosal injury, inflammatory cell infiltration, and crypt damage in the DSS group (Fig. 6I). In contrast, the DSS + BiHANs group exhibited minimal mucosal damage, demonstrating the strong therapeutic efficacy of BiHANs against DSS-induced acute colitis.

Finally, macrophage infiltration was assessed via immunofluorescence staining for F4/80 in colon tissue. As shown in Fig. 6J, BiHANs significantly reduced macrophage infiltration in IBD-affected colon tissue, outperforming colloidal BT. Taken together, these findings highlight the potential of BiHANs as promising candidates for clinical applications in IBD therapy.

### 3.7 Modulation of BiHANs on the gut microbiome by 16S rRNA sequencing analysis

**Fig. 7:**
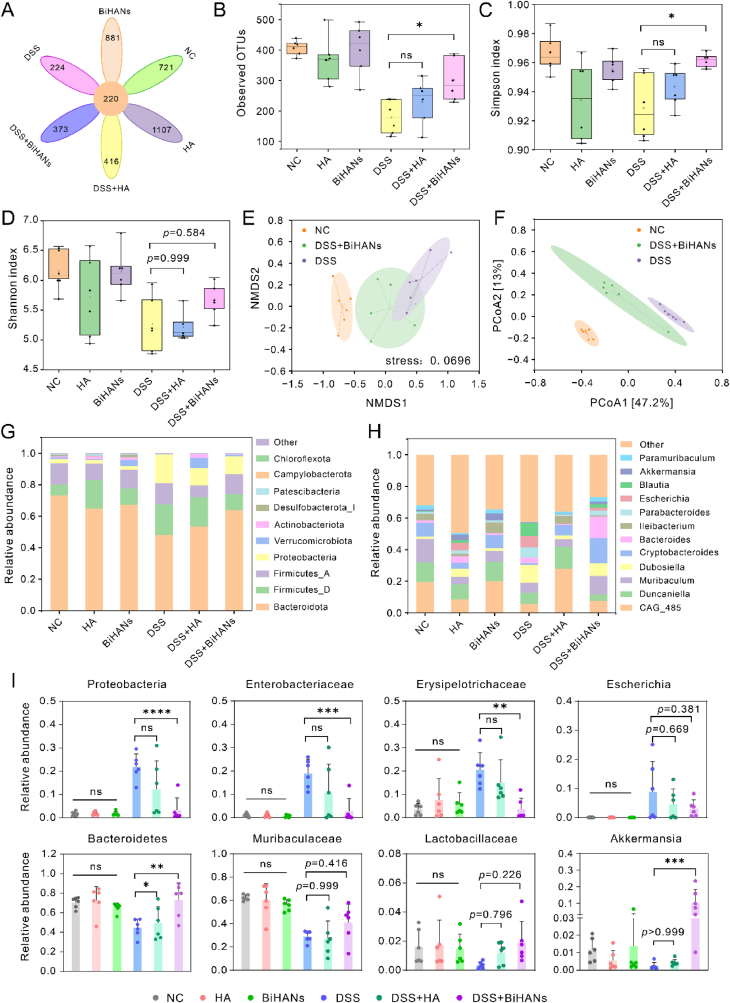
BiHANs modulating the gut microbiota of chronic colitis. A) Petal diagram illustrating the gut microbiota composition in different groups, with values between the circles representing the number of shared species. B, C, D) OTU richness, Simpson index, and Shannon index, showing the diversity of the gut microbiota across the groups. E, F) NMDS and PCoA plots demonstrating the β-diversity of the gut microbiome among the groups. G, H) Relative abundance of the gut microbiota at the phylum and genus levels in the different groups. I) Relative abundances of key harmful and probiotic microbiota in the different groups. Data are presented as mean ± S. D. from a representative experiment (n = 6 biologically independent animals for each group. Data were analyzed by one-way ANOVA with Tukey's multiple comparisons test (B-D, I). *∗p* < 0.05, ∗∗*p* < 0.01, ∗∗∗*p* < 0.001, ∗∗∗∗*p* < 0.0001 and ns represents no statistical difference.

Emerging evidence from preclinical and clinical studies underscores the pivotal role of gut microbiota dysbiosis in the progression of IBD and its impact on treatment efficacy [35,39]. Motivated by the promising therapeutic effects of BiHANs, we explored their potential to ameliorate gut microbiota dysbiosis in a murine IBD model. Fecal samples were collected and subjected to 16S rRNA sequencing to evaluate the composition and abundance of the gut microbiota (Fig. S14). The petal diagram showed that BiHANs significantly enhanced gut microbiome richness, bringing it closer to levels observed in the healthy control group (Fig. 7A).

Analysis of operational taxonomic unit (OTU) richness (Fig. 7B) and α-diversity indices, including the Simpson (Fig. 7C) and Shannon indices (Fig. 7D), revealed a significant reduction in microbiome diversity after DSS treatment. However, BiHANs administration significantly restored these diversity metrics. β-diversity analysis, using nonmetric multidimensional scaling (NMDS) and principal component analysis (PCoA), demonstrated a clear separation between the DSS-treated and healthy groups. Notably, the DSS + BiHANs group clustered more closely with the healthy group, indicating that BiHANs effectively mitigated DSS-induced gut microbiota perturbations (Fig. 7E and F).

Gut microorganisms play a critical role in immune regulation, intestinal barrier maintenance, and pathogen suppression. In healthy individuals, *Firmicutes* and *Bacteroidetes* are the predominant phyla; however, dysbiosis can lead to chronic inflammation and impaired barrier function. We analyzed the bacterial composition at both the phylum and genus levels. Compared to the healthy group, the DSS group exhibited an elevated relative abundance of *Firmicutes* and *Proteobacteria*, alongside a reduced abundance of *Bacteroidetes* at the phylum level. In contrast, the DSS + BiHANs group showed an increased abundance of *Bacteroidetes* and a reduction in *Firmicutes* and *Proteobacteria*, closely resembling the healthy group (Fig. 7G).

At the genus level (Fig. 7H), DSS treatment significantly reduced *Muribaculaceae* and *Cryptobacteroides* abundances while elevating *Dubosiella* and *Escherichia*. BiHANs administration reversed these trends, restoring beneficial taxa (*Muribaculaceae*, *Cryptobacteroides*) and suppressing harmful genera (*Dubosiella*, *Escherichia*), including *Enterobacteriaceae* pathogens linked to gastrointestinal symptoms (Fig. 7I). To establish microbiota-anti-inflammatory crosstalk, Pearson correlation analysis (Fig. S15, Table S4) revealed strong negative associations between pro-inflammatory cytokines (IL-6, TNF-α) and *Enterobacteriaceae* (r = 0.99 and 0.99, respectively; *p* < 0.0001), *Proteobacteria* (r = 0.99 and 1.00; *p* < 0.0001), and *Erysipelotrichaceae* (r = 0.96 and 0.98, *p* < 0.01). while weaker positive correlations were observed with *Lactobacillaceae* (r = −0.92 and −0.95; *p* < 0.01). These findings solidify the mechanistic link between BiHANs-driven microbiota modulation and IBD symptom alleviation.

### 3.8 BiHANs target mitochondria and maintain homeostasis to suppress NF-κB-driven inflammation

**Fig. 8:**
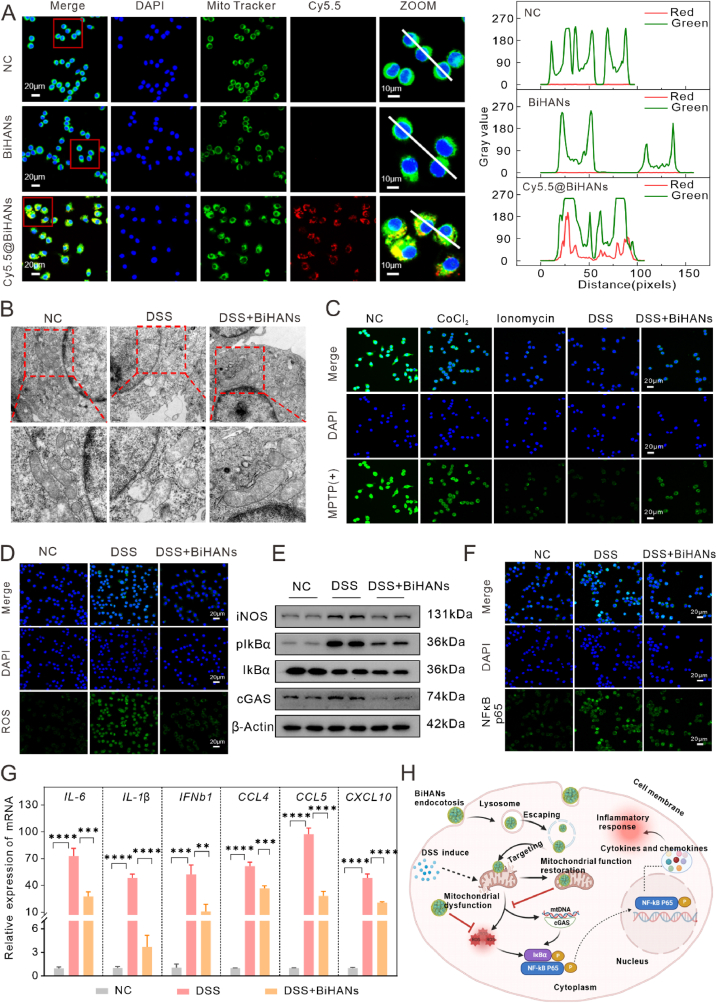
Protective effects and mechanism of BiHANs against DSS-induced inflammatory response in RAW 264.7 cells. A) CLSM image of the colocalization of BiHANs with mitochondria in RAW 264.7 cells and the corresponding fluorescence intensity; scale bar = 10 μm. B) TEM images of mitochondria from different treatment groups of RAW 264.7 cells. C) Fluorescence images of MPTP opening in RAW 264.7 cells in different groups; scale bar = 20 μm. D) Merged images of RAW 264.7 cells subjected to DCFH-DA (a ROS probe) staining (green) and DAPI staining (blue) under different treatment conditions; scale bar = 20 μm. E) WB analysis of the key enzymes regulating inflammation in RAW 264.7 cells with DSS treatment. F) The localization and expression of the p65 protein in RAW 264.7 cells in various groups were observed by immunofluorescence; scale bar = 20 μm. G) RT‒qPCR analysis of the mRNA levels of *IL-6, IL-1β, IFNb1, CCL4, CCL5* and *CXCL10* in RAW 264.7 macrophages. RAW 264.7 macrophages were pretreated with BiHANs (10 μg/mL) for 24 h, followed by stimulation with DSS (2 mg/mL) for another 24 h n = 3. The data are presented as the mean ± S. D. (n = 3). ∗*p* < 0.05, ∗∗*p* < 0.01 and ∗∗∗*p* < 0.001. H) Schematic illustration of the protective effects and mechanism of BiHANs on DSS-induced imflammatory response. Data were analyzed by one-way ANOVA with Tukey's multiple comparisons test (G). (For interpretation of the references to color in this figure legend, the reader is referred to the Web version of this article.)

Despite BiHANs demonstrating efficacy in treating DSS-induced IBD, their underlying therapeutic mechanisms remain incompletely understood. Considering that mitochondrial dysfunction is closely associated with IBD pathogenesis [37], we hypothesized that BiHANs may ameliorate IBD by preserving mitochondrial homeostasis. To validate this hypothesis, we first examined the subcellular localization of BiHANs in RAW 264.7 macrophages using confocal microscopy, which revealed that BiHANs specifically accumulate in mitochondria (Fig. 8A). Building on this finding, we next evaluated whether BiHANs can counteract DSS-induced mitochondrial damage by performing Bio-TEM analysis. The TEM images showed that DSS exposure resulted in severe mitochondrial swelling, cristae disruption, and membrane rupture, whereas BiHANs treatment effectively preserved mitochondrial morphology, maintaining structural integrity comparable to that of control cells (Fig. 8B). These results indicate that BiHANs protect against DSS-induced mitochondrial damage.

To further assess the functional impact of BiHANs on mitochondrial health, we evaluated mitochondrial permeability transition pore (MPTP) opening using Calcein AM/CoCl_2_ staining. Excessive MPTP opening is known to trigger mitochondrial dysfunction and ROS overproduction; therefore, we next measured intracellular ROS levels. BiHANs treatment significantly inhibited MPTP opening, as evidenced by enhanced fluorescence intensity compared to DSS-treated cells (Fig. 8C), and markedly reduced DSS-induced ROS accumulation in RAW 264.7 cells (Fig. 8D), underscoring their potent antioxidant properties and protective role in mitigating oxidative stress.

Given the pivotal role of mitochondrial dysfunction and oxidative stress in activating inflammatory signalling, we further investigated the impact of BiHANs on NF-κB activation. Western blot analysis demonstrated that BiHANs significantly downregulated the expression of inducible nitric oxide synthase (iNOS) and cyclic GMP-AMP synthase (cGAS), while suppressing IκBα phosphorylation, thereby inhibiting NF-κB activation (Fig. 8E). Immunofluorescence analysis confirmed that BiHANs effectively blocked the nuclear translocation of the NF-κB p65 subunit (Fig. 8F). Furthermore, quantitative PCR analysis revealed that BiHANs significantly reduced the mRNA expression of pro-inflammatory cytokines (IL-6 and IL-1β) and chemokines (IFNB1, CCL4, CCL5, and CXCL10) in DSS-treated RAW 264.7 cells (Fig. 8G).

Taken together, these findings suggest that BiHANs exert their anti-inflammatory effects, at least in part, by targeting mitochondria, preserving mitochondrial homeostasis, reducing oxidative stress, and suppressing NF-κB signalling, thereby mitigating intestinal inflammation in IBD.

## 4 Discussion

The findings presented in this study demonstrate the multifaceted therapeutic potential of BiHANs in the treatment of IBD. Through a combination of *in vitro* and *in vivo* experiments, we have elucidated the mechanisms by which BiHANs exert their antioxidant, anti-inflammatory, and microbiota-modulating effects, ultimately leading to the alleviation of IBD symptoms.

One of the key findings is the ability of BiHANs to target mitochondria and maintain mitochondrial homeostasis, which is crucial given the central role of mitochondrial dysfunction in IBD pathogenesis [[40], [41], [42]]. The confocal microscopy and TEM analyses revealed that BiHANs effectively localize within mitochondria, restoring mitochondrial morphology and function in DSS-treated cells. This mitochondrial targeting capability, coupled with the significant reduction in ROS levels and inhibition of MPTP opening, underscores the potent antioxidant activity of BiHANs. These results are consistent with previous studies highlighting the importance of mitochondrial integrity in mitigating inflammation and oxidative stress in IBD [37].

Furthermore, BiHANs demonstrated a remarkable ability to modulate the NF-κB signaling pathway, a critical regulator of inflammation [[43], [44], [45]]. By downregulating the expression of pro-inflammatory cytokines such as TNF-α, IL-1β and IL-6, and inhibiting the nuclear translocation of NF-κB p65, BiHANs effectively suppressed the inflammatory response in both *in vitro* and *in vivo* models. This dual modulation of mitochondrial function and NF-κB signaling provides a mechanistic basis for the observed therapeutic efficacy of BiHANs in alleviating colitis.

In addition to their anti-inflammatory and antioxidant properties, BiHANs were shown to significantly modulate the gut microbiota, restoring microbial diversity and promoting the growth of beneficial bacteria while suppressing harmful species. The 16S rRNA sequencing analysis revealed that BiHANs treatment led to an increase in the abundance of *Bacteroidetes* and a reduction in *Firmicutes* and *Proteobacteria*, closely resembling the microbial profile of healthy controls. This restoration of gut microbiota homeostasis is particularly important given the well-established link between dysbiosis and IBD progression [32]. Future studies should integrate metagenomic/metabolomic profiling to dissect functional pathways (e.g., short-chain fatty acid synthesis) mediated by BiHANs-modulated microbiota, validate causal relationships via fecal microbiota transplantation in germ-free models, and employ *in vitro* coculture systems to clarify direct effects of BiHANs on critical taxa such as *Muribaculaceae* and *Escherichia*.

The *in vivo* studies further corroborated the therapeutic potential of BiHANs, demonstrating their ability to accumulate specifically in inflamed colon tissues, as evidenced by CT and fluorescence imaging. This targeted accumulation, combined with the lack of systemic toxicity, highlights the clinical translatability of BiHANs as a safe and effective treatment for IBD. Moreover, the comparative efficacy studies revealed that BiHANs outperformed the clinically established bismuth tartrate (BT) in mitigating colitis symptoms, further underscoring their superior therapeutic profile. Scalability analyses suggest feasible production via microfluidic systems (>50 % cost reduction) with stable bioactivity, though lyophilization protocols require further optimization for clinical translation. Future pharmacokinetic studies employing radiolabeling and ICP-MS will precisely map BiHANs' biodistribution and clearance pathways (renal/hepatic), while GLP-compliant toxicology assessments (28-day repeat-dose in swine) and minipig colitis models will validate targeting specificity.

Importantly, BiHANs represent a novel formulation of humic acid (HA), significantly enhancing its therapeutic efficacy. By chelating bismuth ions (Bi^3+^) with HA, BiHANs not only improve the stability and bioavailability of HA but also endow it with additional functionalities, such as mitochondrial targeting and enhanced antioxidant activity. This innovative formulation approach provides a new direction for the development of HA-based therapeutics, addressing some of the limitations associated with traditional HA applications. The selection of Bi^3+^ as the coordinating ion was strategically driven by its mucosal-protective properties, low systemic absorption risks, and minimized oxidative toxicity compared to redox-active metals like Fe^3+^ or cytotoxic Zn^2+^ [[46], [47], [48]], aligning with clinical precedents of bismuth-based gastrointestinal therapies while overcoming their coordination instability.

Moreover, the success of BiHANs as a novel nanotherapeutic platform offers valuable insights for the development of new formulations for complex natural product systems, such as traditional Chinese medicine (TCM) decoctions. Many TCM formulations contain multiple active components with poor solubility, stability, or bioavailability, limiting their clinical application. The self-assembly strategy employed in BiHANs, which leverages the coordination between metal ions and natural polymers, could serve as a reference for designing advanced delivery systems for TCM and other complex natural product mixtures. This approach could enhance the therapeutic efficacy of these formulations while maintaining their safety profile. Additionally, the strong X-ray attenuation capability of BiHANs, attributed to the high atomic number of bismuth, suggests their potential as CT contrast agents, further expanding their clinical applications.

In conclusion, this study provides compelling evidence that BiHANs represent a promising therapeutic strategy for IBD. By targeting mitochondrial dysfunction, modulating the NF-κB pathway and restoring gut microbiota balance, BiHANs address multiple facets of IBD pathogenesis. Furthermore, BiHANs exemplify a new formulation strategy for enhancing the therapeutic potential of humic acid and offer a blueprint for the development of advanced delivery systems for complex natural product mixtures, including TCM decoctions (Scheme 1B). These findings pave the way for further clinical development of BiHANs as a novel, multifunctional nanotherapeutic for IBD and potentially other inflammatory diseases. The enhanced efficacy of HA in this new formulation, along with its potential as a CT contrast agent, opens new avenues for both therapeutic and diagnostic applications.

## CRediT authorship contribution statement

**Ganglin Wang:** Writing – review & editing, Writing – original draft, Supervision, Project administration, Methodology, Investigation, Funding acquisition, Formal analysis, Data curation, Conceptualization. **Ziwei Wang:** Visualization, Software, Methodology, Investigation, Formal analysis, Data curation. **Lin Liu:** Writing – original draft, Supervision, Project administration. **Yejing Zhu:** Formal analysis, Data curation. **Jiali Zhong:** Formal analysis, Data curation. **Jiayi Zhang:** Formal analysis, Data curation. **Lingling Wang:** Formal analysis, Data curation. **Chenguo Zheng:** Supervision, Resources, Conceptualization. **Wei Li:** Writing – review & editing, Supervision, Project administration, Funding acquisition, Conceptualization.

## Declaration of competing interest

The authors declare no competing financial interests or personal relationships that could influence the work reported in this paper. The research was financially supported by the 10.13039/501100004731Natural Science Foundation of Zhejiang Province (Grant No. Y24H030041), the 10.13039/501100001809National Natural Science Foundation of China (Grant No. 81970753), and the 10.13039/501100007194Wenzhou Science and Technology Bureau Foundation (Grant No. ZY2021011). These funding sources not been involved in the study design, data collection, analysis, interpretation, manuscript preparation, or decision to submit the article for publication.

No patents or commercial applications related to the bismuth-humic acid nanoparticles (BiHANs) described in this study are currently pending or under development. All authors confirm that they have no affiliations with or involvement in any organization or entity with any financial or non-financial interest in the subject matter or materials discussed in this manuscript.

Animal experiments were conducted in strict compliance with the ethical guidelines approved by the Wenzhou Medical University Animal Care and Use Committee (Approval No. xmsq2024-0205). All data supporting the findings of this study are available from the corresponding author upon reasonable request, with no restrictions imposed by funding agencies or institutional policies.

## Data Availability

Data will be made available on request.
