# Supplementary material for: Alchemizing earth's legacy: Bismuth-engineered humic nanoparticles for IBD theranostics through mitochondrial anti-inflammation and sustained intestinal delivery
Source: Mater Today Bio. 2025 Jun 6;33:101948. doi: 10.1016/j.mtbio.2025.101948 (PMC12181014; doi:10.1016/j.mtbio.2025.101948)
Supplement: Multimedia component 1 [file mmc1.docx]

Supporting Information

Alchemizing Earth's Legacy: Bismuth-Engineered Humic Nanoparticles for IBD Theranostics Through Mitochondrial Anti-Inflammation and Sustained Intestinal Delivery

*Ganglin Wang^a, †^, Ziwei Wang ^a, †^, Lin Liu ^a, †^, Yejing Zhu ^a^, Jiali Zhong ^a^, Jiayi Zhang ^a^, Lingling Wang ^a^, Chenguo Zhen ^b, *^ and Wei Li ^a, *^*

a. Key Laboratory of Laboratory Medicine, Ministry of Education of China, Zhejiang Provincial Key Laboratory of Medical Genetics, School of Laboratory Medicine and Life Sciences, Wenzhou Medical University, Wenzhou 325035, China

b. Department of Coloproctology, The Second Affiliated Hospital and Yuying Children’s Hospital of Wenzhou Medical University, Wenzhou 325000, PR China

^†^ These authors contributed equally to this work.

Email: liweiwzmc@163.com, zhengchenguo80@163.com


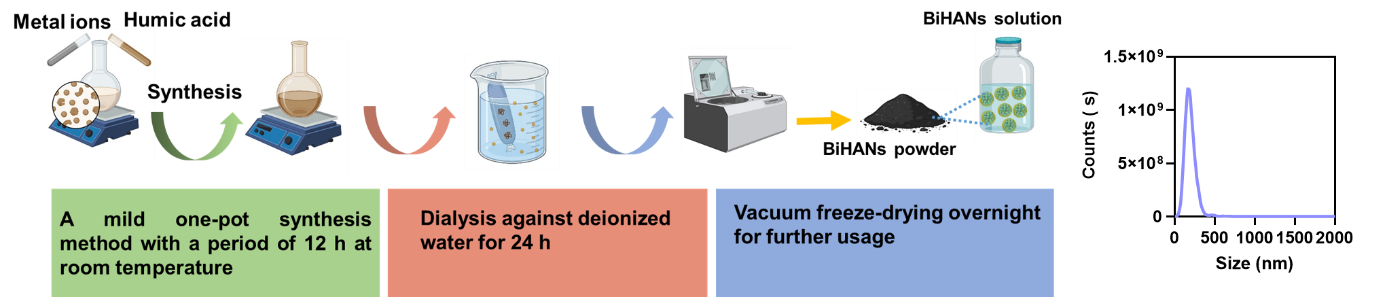
**Supporting Figures and Tables**

**Fig. S1** Schematic illustration of the synthesis and purification of BiHANs.


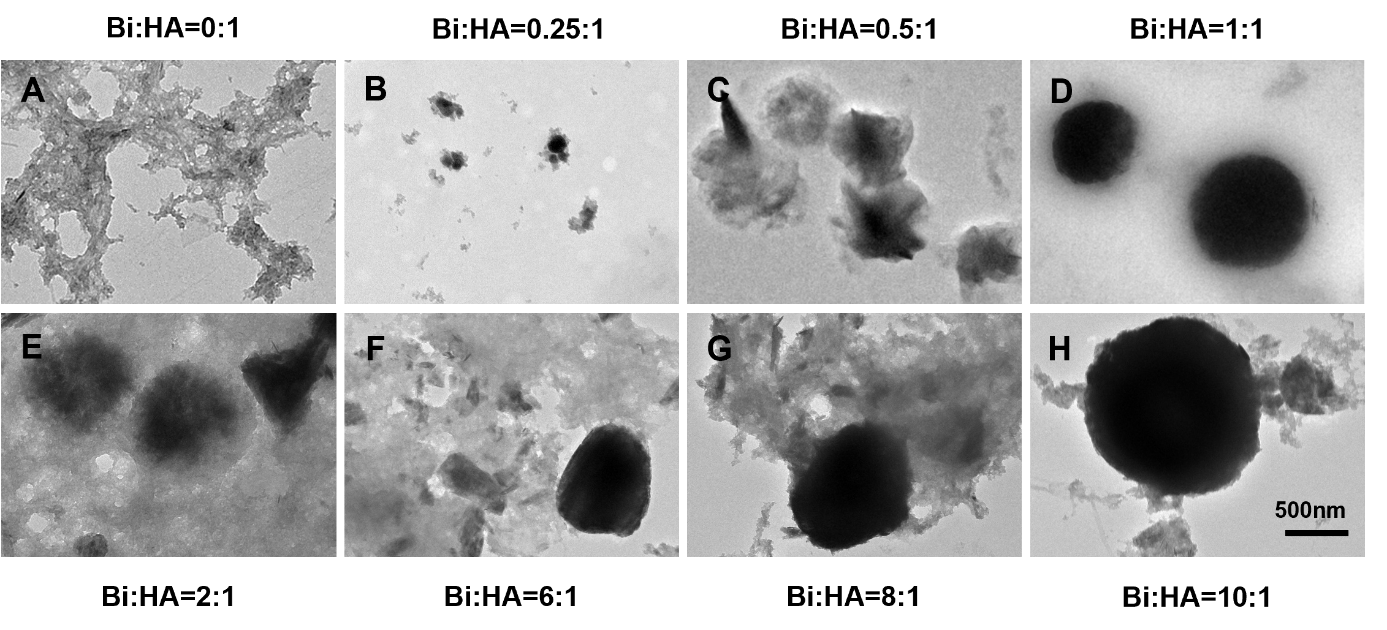


**Fig. S2** Transmission electron microscope (TEM) images of BiHANs prepared at different feeding of mass ratios of Bi^3+^ and HA. Bi^3+^: HA= 0:1 (A), Bi^3+^: HA= 0.25:1 (B), Bi^3+^: HA= 0.5:1 (C), Bi^3+^: HA= 1:1 (D), Bi^3+^: HA= 2:1 (E), Bi^3+^: HA= 6:1 (F), Bi^3+^: HA= 8:1 (G), Bi^3+^: HA= 10:1 (H).

***Fig. S3*** Size distribution of BiHANs by Dynamic light scattering (DLS).


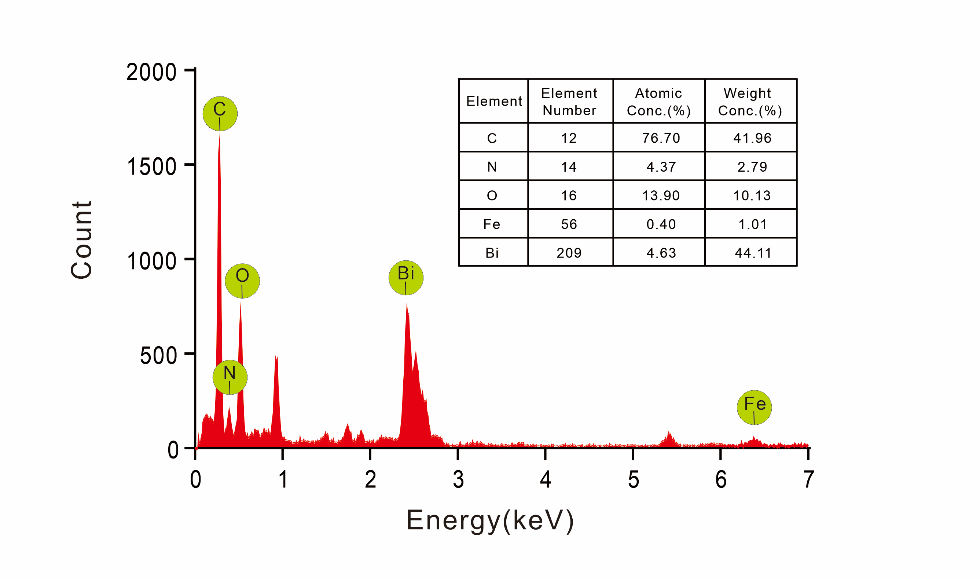


**Fig. S4** Energy dispersive spectrum of BiHANs and the accompanying table presented a comprehensive analysis of the elemental composition in the prepared BiHANs.


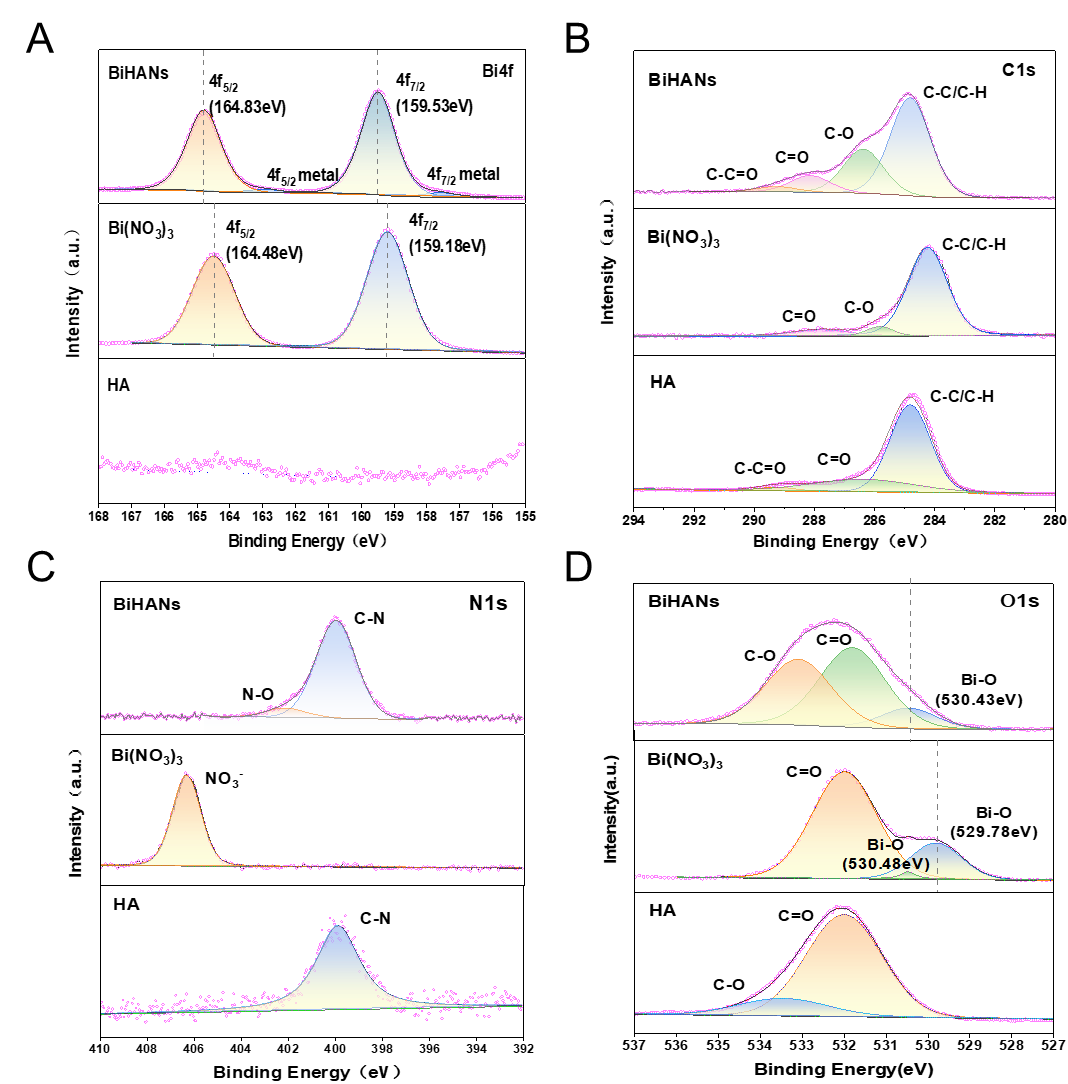


**Fig. S5** XPS spectrum of Bi4f (A), XPS spectrum of C1s (B), XPS spectrum of N1s (C), XPS spectrum of O1s (D) in BiHANs, Bi(NO_3_)_3_ and HA_._


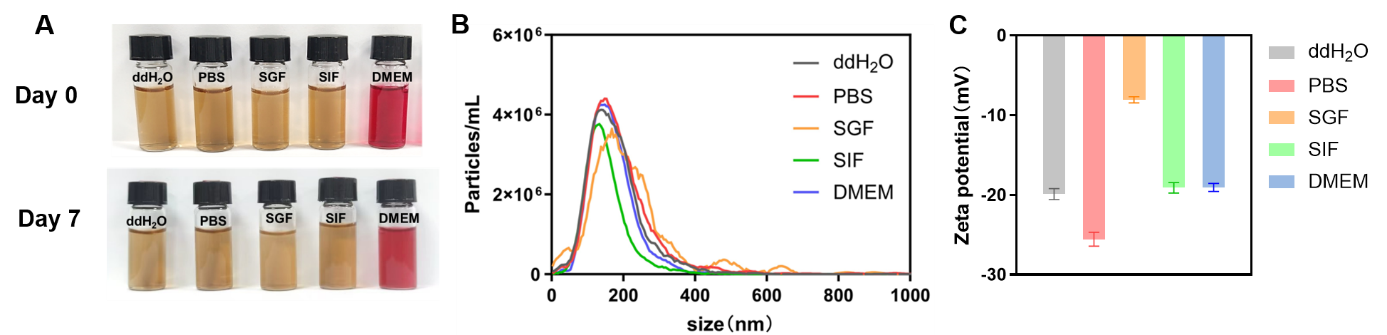


**Fig. S6** Long-term colloidal stability of BiHANs in different media (form left to right in each photo were water, PBS, SGF, SIF and DMEM, respectively) for 0 and 7 days (A). Size distribution of BiHANs in different media after 7 days incubation (B). Zeta potential of BiHANs in different media after 7 days incubation (C).


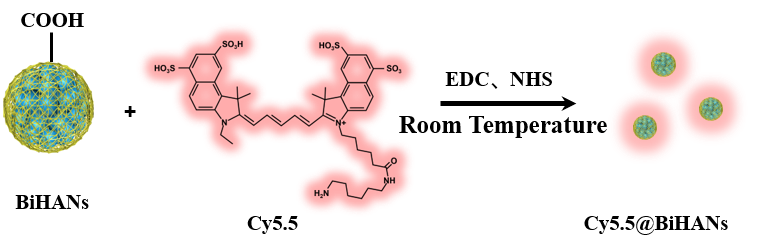


A

B

C


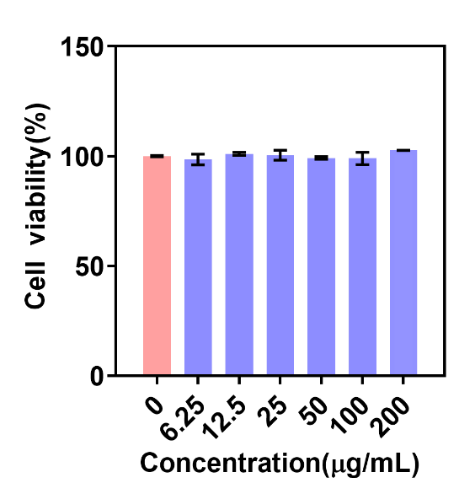

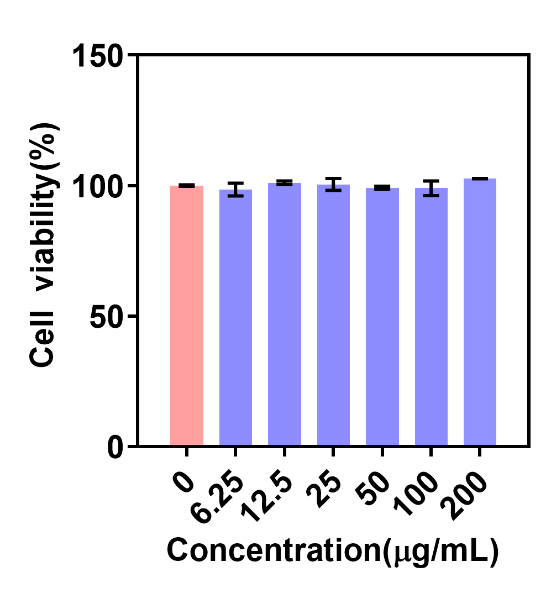


**Fig. S7** Synthesis of Cy5.5@BiHANs (A). Cytotoxicity of Cy5.5@BiHANs on RAW 264.7 cells and Caco2 cells.


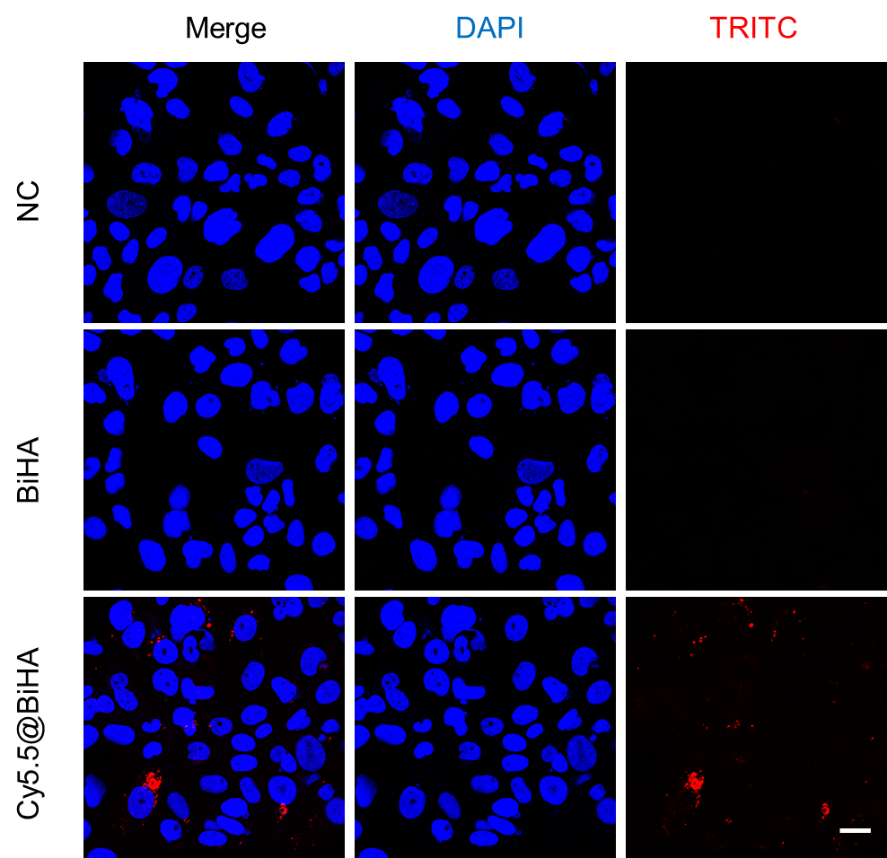


**Fig. S8** Cellular uptake profiles by the fluorescence images of Caco2 cells treated with 20 µg/mL of BiHANs and 20 µg/mL of Cy5.5@BiHANs for 24 h, respectively. Scale bar: 25 μm.

A

B


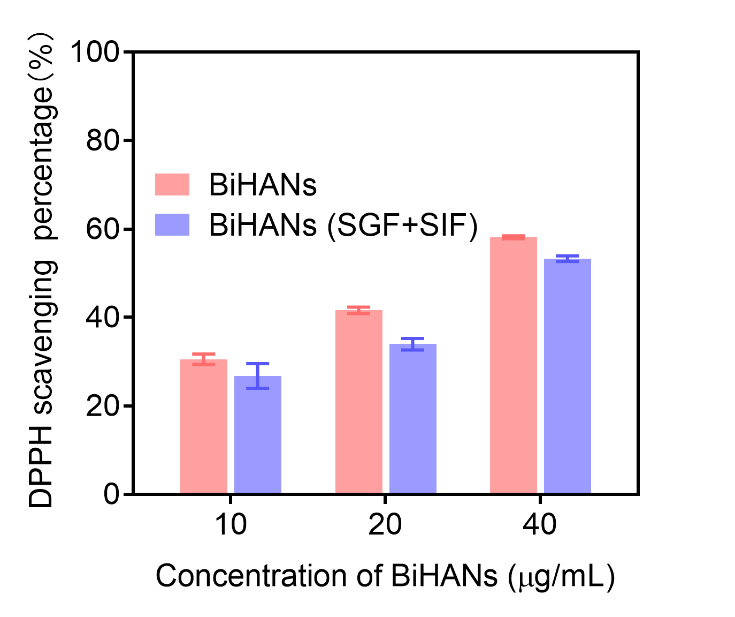

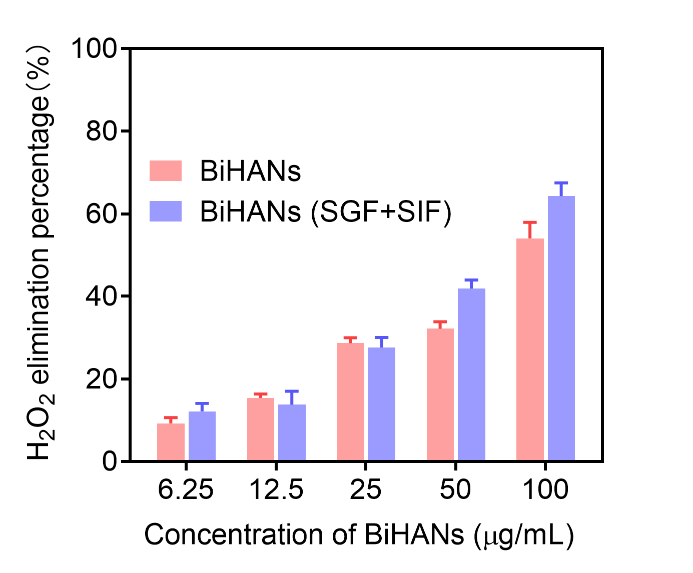


**Fig. S9** ROS-scavenging ability of BiHANs in SGF+SIF treatment, of which BiHANs were treated with SGF for 2h, followed by SIF for 4h. The elimination efficiency of BiHANs with different concentrations after SGF+SIF treatment by peroxy radicals generated from 1 mM H_2_O_2_ (A). DPPH scavenging ability of BiHANs with different concentrations after SGF+SIF treatment (B).


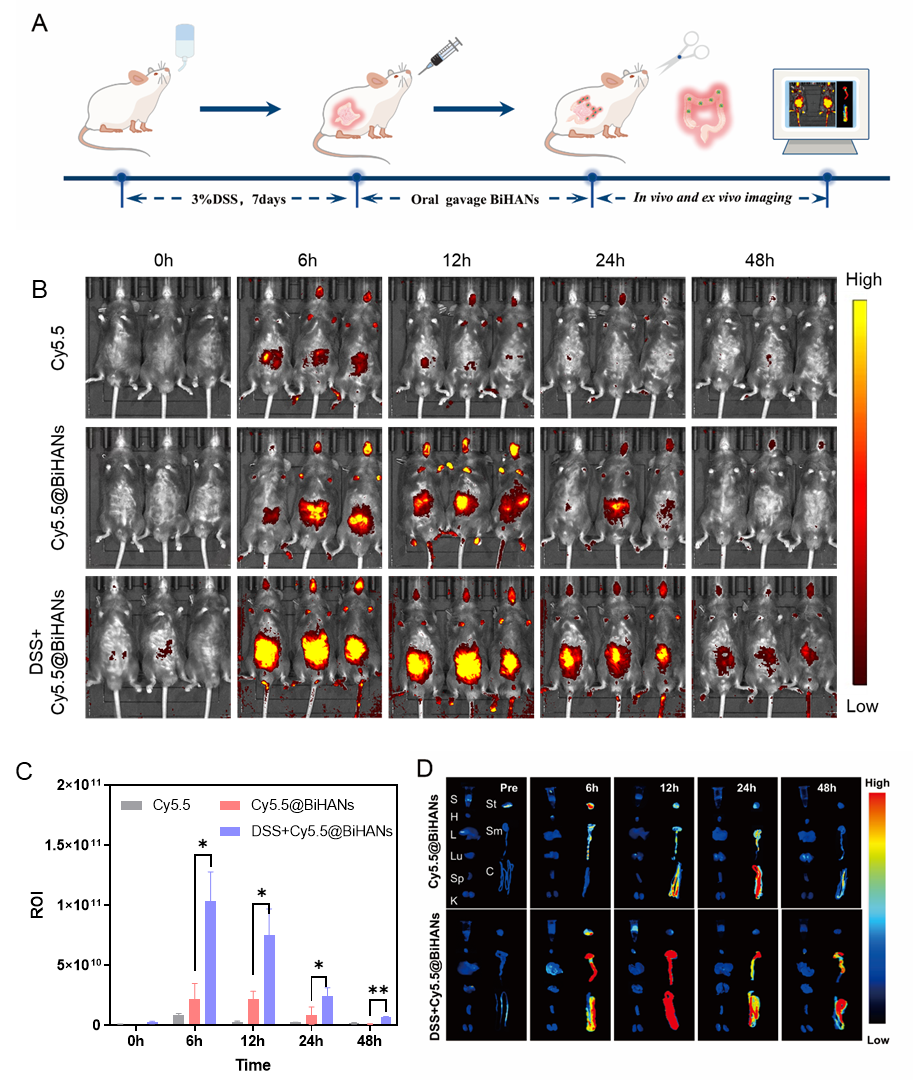


**Fig. S10** Schematic illustration of experimental protocol for *in vivo* and *ex vivo* fluorescence imaging of Cy5.5@BiHANs in mice (A). Time-dependent of *in vivo* fluorescent images of representative healthy and colitis model mice which were orally administered administration of Cy5.5@BiHANs, imaged by an *in vivo* imaging system (IVIS) (B, C). Time-dependent of *ex vivo* fluorescent images of representative healthy and colitis model mice which were orally administered administration of Cy5.5@BiHANs, imaged by an in vivo imaging system (IVIS), S: serum; H: heart; L: liver; Lu: lung; Sp: spleen; K: kidney; St: stomach; Sm: small intestine; C: colon (D). Data are presented as mean ± S. D. Representative images or quantitative analyses of n = 3 animals from two independent experiments [(B) to (D)] are shown. The data are presented as the means ± S. D. * *p*< 0.05, ** *p* < 0.01, *** *p* < 0.001; the statistical significance was analyzed using unpaired Student’s two-tailed t-test.


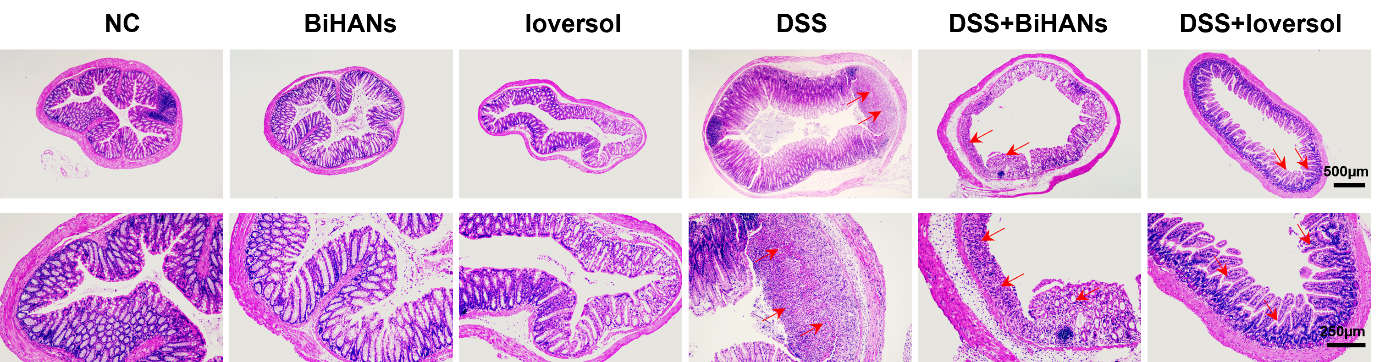


**Fig. S11** H&E-stained colonic sections corresponded with Fig. 4D.


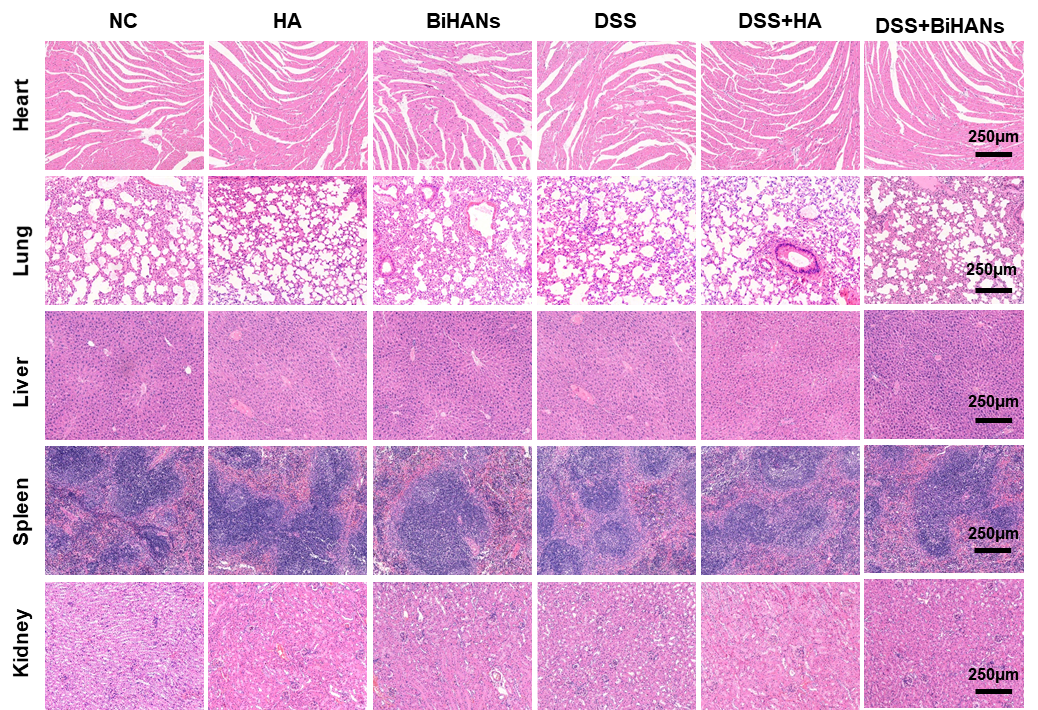


**Fig. S12** H&E staining of major organs of mice in each group. The representative images are shown from three independent mice.


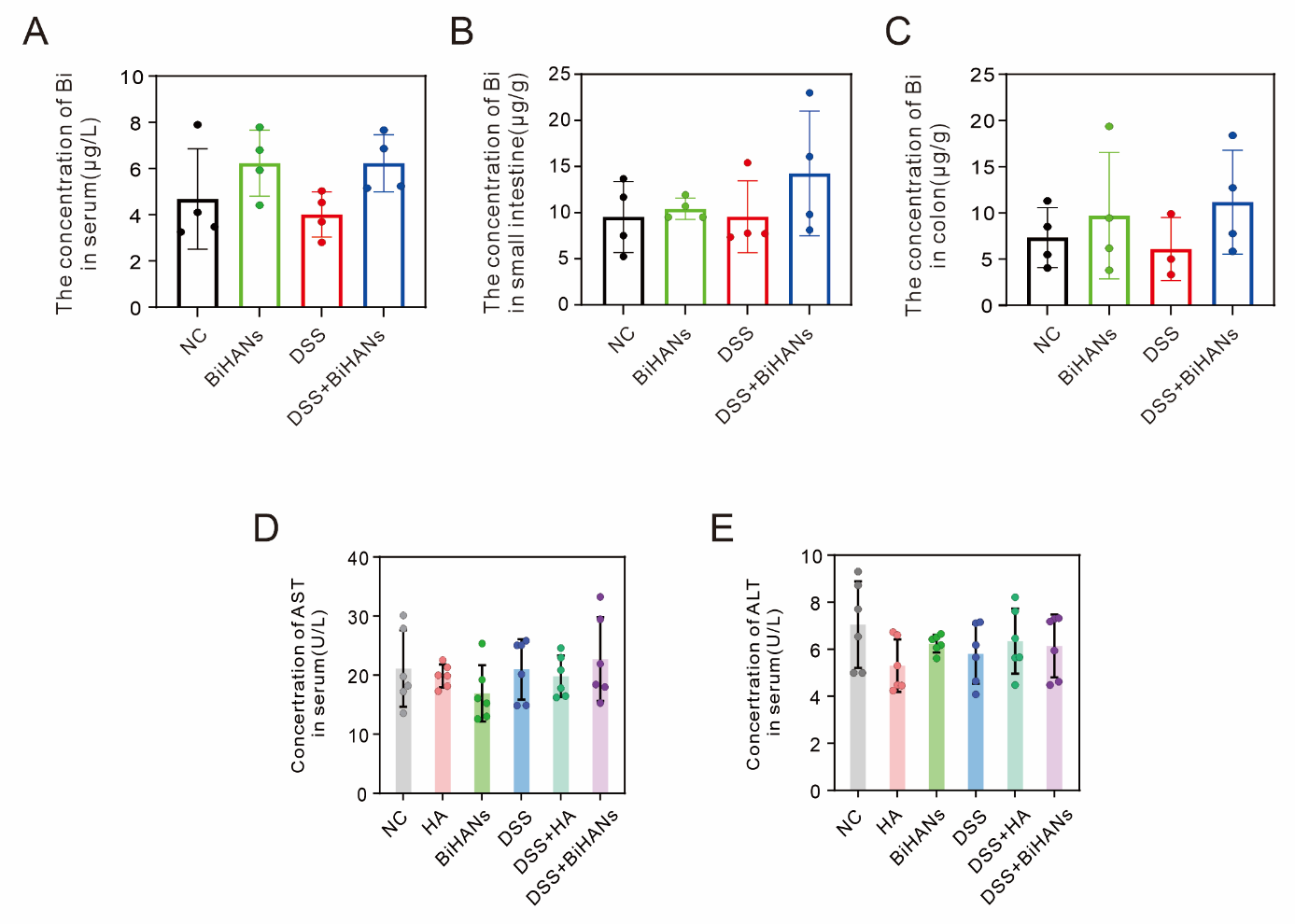


**Fig. S13** Biodistribution profile of BiHANs in the serum of mice in each group (A). Biodistribution profile of BiHANs in the small intestine of mice in each group (B). Biodistribution profile of BiHANs in the colon of mice in each group (C). The routine blood index test (D and E).


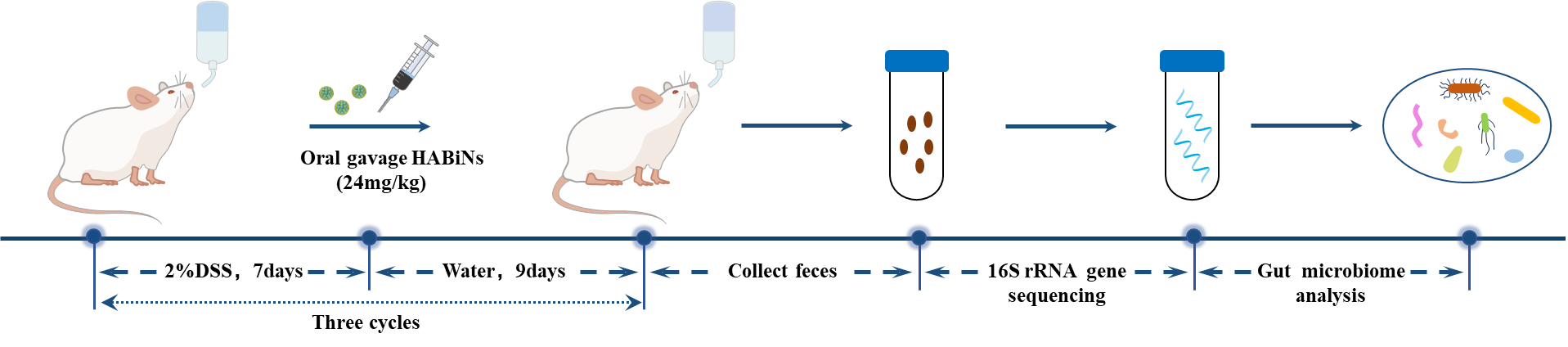


**Fig. S14** Schematic illustration of 16S rRNA sequencing to evaluate the composition and abundance of gut microbiota mice.


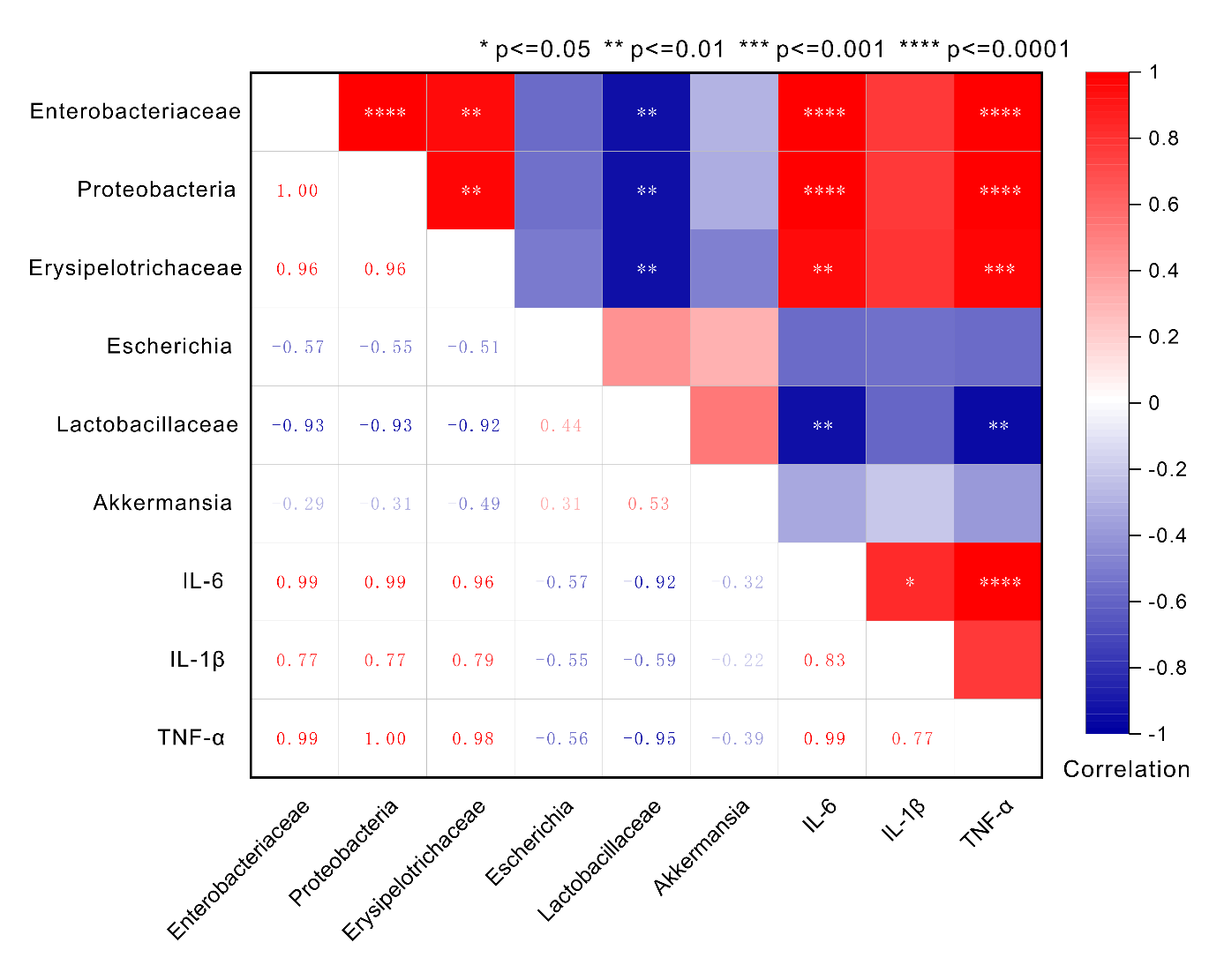


**Fig. S15** Heatmap visualizing significant Pearson correlations (*p*<0.05) between gut microbiota composition and proinflammatory cytokines (IL-6, IL-1β, TNF-α). The color scale bar ranged from −1.0 (blue) to 1.0 (red). The number and color of the squares indicate the magnitude of the correlation between parameters. Red and blue, respectively, denote positive and negative correlations.

***Table S1.*** Genes and as sociated primer sequences used for RT-qPCR analysis.

| Gene |  | Primer sequence |
| --- | --- | --- |
| *GAPDH* | Forward | 5'-AGGTCGGTGTGAACGGATTTG-3' |
|  | Reverse | 5'-GGGGTCGTTGATGGCAACA-3' |
| *IL-6* | Forward | 5'-CGGCCTTCCCTACTTCACAA-3' |
|  | Reverse | 5'-TTGCCATTGCACAACTCTTTTC-3' |
| *IL-1β* | Forward | 5'-GCAACTGTTCCTGAACTCAACT-3' |
|  | Reverse | 5'-ATCTTTTGGGGTCCGTCAACT-3' |
| *TNF-α* | Forward | 5'-GACGTGGAACTGGCAGAAGAG-3' |
|  | Reverse | 5'-TTGGTGGTTTGTGAGTGTGAG-3' |

***Table S2.*** Average hemolysis rates 3h after incubation with BiHANs.

| Concentration(µg/mL) | Hemolysis（%） |
| --- | --- |
| PBS | 0 |
| ddH_2_O | 100 |
| 25 | 0.528 |
| 50 | 1.515 |
| 75 | 3.078 |
| 100 | 4.595 |
| 200 | 5.660 |

***Table S3.*** Disease Activity Index of colitis in C57BL/6J mice.

| Score | Weight loss(%) | Stool consistency | Occult blood |
| --- | --- | --- | --- |
| 0 | 0 | Normal | Negative |
| 1 | 1~5 | Soft | Weak positive |
| 2 | 5~10 | Loose | Positive |
| 3 | 10~20 | Mushy | Strong positive |
| 4 | >20 | Watery diarrhea | Gross perianal bleeding |

***Table S4.*** Pearson correlations (r value) between gut microbiota composition and proinflammatory cytokines (IL-6, IL-1β, TNF-α). ***p*<0.01, ****p*<0.001, *****p*<0.0001.

| Indicators | IL-6 | IL-1β | TNF-α |
| --- | --- | --- | --- |
| Enterobacteriaceae | 0.99**^****^** | 0.77 | 0.99**^****^** |
| Proteobacteria | 0.99**^****^** | 0.77 | 1.00**^****^** |
| Erysipelotrichaceae | 0.96**^**^** | 0.79 | 0.98**^***^** |
| Escherichia | -0.57 | -0.55 | -0.56 |
| Lactobacillaceae | -0.92**^**^** | -0.59 | -0.95**^**^** |
| Akkermansia | -0.32 | -0.22 | -0.39 |
